# Surface Observables, 2-Knot Invariants, and Nonabelian Electric Fluxes

**DOI:** 10.1007/s00220-026-05725-y

**Published:** 2026-09-27

**Authors:** Alberto S. Cattaneo

**Affiliations:** Institut für Mathematik, Universität Zürich, Winterthurerstrasse 190, CH-8057 Zürich, Switzerland

## Abstract

This work introduces a surface observable for nonabelian four-dimensional *BF* theory with a cosmological term. The surface observable yields new 2-knot invariants that may extend beyond known examples such as the Alexander invariant. By BV pushforward, the surface observable induces an electric observable in nonabelian Yang–Mills theory, offering a concrete realization of ’t Hooft operators. An application to self-dual Yang–Mills theory is also discussed.

## Introduction

The main goal of this paper is to introduce a surface observable for *BF* theory with cosmological term in four dimensions and to use it to define 2-knot invariants and an electric observable for nonabelian Yang–Mills theory. We shortly recapitulate the two independent parts.

### The electric observable

Recall that in abelian Yang–Mills theory (i.e., in electromagnetism), the curvature $F_A={\textrm{d}}A$ is gauge invariant. As a result, we can consider observables like $\int _\Sigma F_A$ (magnetic flux) and $\int _\Sigma *F_A$ (electric flux). Here ∗ is the Hodge dual by the background metric (which in this paper we assume to be riemannian in order to have a decomposition of 2-forms into self dual and anti self dual ones).

If Σ has a boundary γ, then, in the abelian case, $\int _\Sigma F_A=\int _\gamma A$, so $\exp \left( {\textrm{i}}\lambda \int _\Sigma F_A\right)$ may be understood as a Wilson loop along γ, which makes sense also in the nonabelian case. If Σ is closed, a nonabelian version of $\exp \left( {\textrm{i}}\lambda \int _\Sigma F_A\right)$ was introduced in [2].

The goal of this paper is to define a nonabelian electric observable, by correcting the abelian one by suitable ħ-corrections:

$$ \mathcal {O}_\Sigma = \textrm{e}^{\frac{{\textrm{i}}}{4\lambda }\int _\Sigma \langle \xi , *F_A\rangle +\, \cdots }. $$

Here λ is related to the Yang–Mills coupling constant, $\langle \, , \, \rangle$ is an invariant pairing on the Lie algebra (e.g., the Killing form if we work with a semisimple, compact Lie group), and ξ is a chosen element of the Lie algebra. In this paper, we work perturbatively, so ξ can be anything. However, we expect that ξ should eventually belong to a prequantizable coadjoint orbit.^1^

These electric observables may be an explicit realization of those proposed by ’t Hooft [71] which play a preeminent role in the modern relation between topological operators and symmetry [44, 47] and may be regarded as topological defects (see, e.g., among others [5, 15, 42, 46, 63] and references therein).^2^

### The surface observable and 2-knot invariants

The starting point of our construction is a surface observable for *BF* theory with cosmological term. This is a generalization of the surface observable for pure *BF* theory introduced in [33]. As the latter, this observable is defined in terms of additional surface fields. Related constructions appear in [8, 62] and in [2, 60, 64]. Unlike the observables proposed in [21, 23, 37], which required to view the surface as a loop of loops, this observable is intrinsically defined in terms of the surface with no additional structure.

Its explicit expression as well as its expectation value can be written in terms of integrals over compactified configuration spaces à la Fulton–MacPherson–Axelrod–Singer [6, 7, 43] and yields an invariant up to isotopy of the embedded surface (in this paper, like in [33], we focus on the case of a long 2-knot in ${\mathbb {R}}^4$, but the construction is more general).

In the pure case, the invariants produced in [33, 66] contain, as a special case, the one proposed by Bott in [16], have been proved by Watanabe [72] (see also [53, 54]) to be related to the Alexander invariant, and should be related to the invariants introduced by Bar-Natan in [10]. The new invariants introduced in this paper might be independent and deserve further study.

### The BV formalism and the BV pushforward

The main technique to produce the surface observable, as a quantum Batalin–Vilkovisky (BV) observable, is a technique, pioneered in [33] and [55] (see also [27, 29, 35, 36, 58, 59]), called BV pushforward. It allows for integrating out some additional fields, like the surface fields we introduce to define the surface observable.

The same technique may be used to pass from one field theory to another (often producing an equivalence). In this paper, we rely on the equivalence (in the appropriate sense) between four-dimensional *BF* theory with cosmological term and four-dimensional Yang–Mills theory (with quantum corrections) devised in [13]. (This is a sort of four-dimensional version of the exact relationship between *BF* and Yang–Mills theory in two dimensions [74].)

This construction allows us to turn the surface observable for *BF* theory into an electric observable for Yang–Mills theory. A similar construction allows for the construction of surface observables in self-dual Yang–Mills theory.

The surface observable constructed in this paper might ultimately also allow us to construct a surface observable for four-dimensional gravity, using results of [26] and references therein. We defer this to future work.

### Plan of the paper

In Sect.  2 we recall classical *BF* theory with cosmological term in four dimensions and heuristically construct a surface observable. In Sect.  3, we recall the basics of the BV formalism and of the BV pushforward. This section may be skipped by the reader already familiar with these topics. Section 4 is the core of the paper: there we introduce the surface observable and discuss its properties, first at a very heuristic “on shell” level, then more precisely using the formalism of the BV pushforward. In Sect.  5, we describe the Feynman-diagram integration of the surface fields, producing a surface observable for *BF* theory as a functional of the *BF*-theory BV fields only. Sect.  6 contains the first application: the construction of 2-knot invariants. In this section we show (see Sect.  6.1.2) that we really get a quantum BV surface observable for *BF* theory (with a proof up to regularization issues for the BV Laplacian) and we prove (see Sect.  6.1.4) that its expectation value plus a universal correction due to anomalies is an isotopy invariant. Section 7 contains the second application: the construction of an electric observable for nonabelian Yang–Mills theory. Finally, Sect.  8 applies the construction to the simpler case of self-dual Yang–Mills theory.

## The Classical Theory

In this section we analyze the classical theory and its surface observable, showing that it is well-defined on shell. This is intended to give an intuitive understanding of the matter which will be refined and made precise via the BV formalism in the next sections.

### The action

Our theory is *BF* theory in four dimensions. The data are an oriented four-manifold^3^*M* and a *G*-principal bundle P→M. The fields are a connection 1-form *A* and a 2-form *B* in the adjoint bundle $\textrm{ad}P$. In the simplified case when *P* is trivial, we view *A* and *B* as elements of $\Omega ^1(M)\otimes {\mathfrak {g}}$ and of $\Omega ^2(M)\otimes {\mathfrak {g}}$, respectively, where ${\mathfrak {g}}$ is the Lie algebra of *G*. Finally, we assume that the Lie algebra ${\mathfrak {g}}$ is unimodular and is endowed with a nondegenerate invariant pairing $\langle \, , \, \rangle$.

Pure *BF* theory is described by the action functional $S[A,B]:=\int _M BF_A$, where $F_A$ is the curvature of *A* ($F_A={\textrm{d}}A+\frac{1}{2}[A,A]$), and the pairing between the sections of the adjoint bundle (or of the ${\mathfrak {g}}$-valued fields) is understood (we prefer to avoid the more pedantical notation $\langle B,F_A\rangle$ instead of $BF_A$).^4^

In this paper we use an extension of pure *BF* theory involving the “cosmological term” (so called because of its relation with the cosmological term in gravity) $\langle B,B\rangle$, which we will simply write *BB*:

2.1 $$\begin{aligned} S[A,B]:=\int _M \left( BF_A-\frac{\lambda }{2}BB\right) , \end{aligned}$$

where λ is a parameter (with λ=0 we recover the pure theory).

#### Remark 2.1

From now on, whenever two $\textrm{ad}$-sections (or ${\mathfrak {g}}$-valued fields) are juxtaposed, the pairing $\langle \, , \, \rangle$ is understood.^5^

#### Remark 2.2

The minus sign in front of λ is purely conventional (we prefer to put a minus sign here so as to have a plus sign in (2.4) instead of the other way around).

By taking a variation of the action, we see that the Euler–Lagrange (EL) equations are

2.2 $$\begin{aligned} F_A=\lambda B\quad \text {and}\quad {\textrm{d}}_AB=0. \end{aligned}$$

Note that, by the Bianchi identity, the second equation is redundant as long as $\lambda \not =0$.

The theory has the infinitesimal symmetries

2.3a $$\begin{aligned} \delta _{\gamma ,\theta } A&= -{\textrm{d}}_A \gamma - \lambda \theta ,\end{aligned}$$

2.3b $$\begin{aligned} \delta _{\gamma ,\theta } B&= -{\textrm{d}}_A\theta + [\gamma ,B], \end{aligned}$$

where the generators γ and θ are $\textrm{ad}$-valued 0- and 1-forms, respectively. (Also here the choice of signs is purely conventional. It is made in such a way that the signs will disappear in (4.3) below.)

A classical observable is a function of the fields *A* and *B* that is invariant under the transformations (2.3). An on-shell observable is a function that is invariant upon using the EL Eq. (2.2).

### The surface observable

Consider an oriented 2-manifold Σ and a map $\sigma :\Sigma \rightarrow M$. (In the quantum theory, σ will have to be an embedding to avoid singularities in the expectation value of the observable we are going to introduce; however, at this level we can work with any map σ.)

We can use σ to pull back the adjoint bundle $\textrm{ad}P$ as well as the fields *A* and *B*. For simplicity of notation, we will keep writing *A* and *B* instead of $\sigma ^*A$ and $\sigma ^*B$. We now introduce new fields on Σ, both taking values in $\sigma ^*\textrm{ad}P$: a 1-form α and a 0-form β. We then consider the following version of two-dimensional *BF* theory coupled to *A* and *B* as external sources:

2.4 $$\begin{aligned} J[\alpha ,\beta ;A,B] :=\int _\Sigma \left( \beta \,{\textrm{d}}_A\alpha +\frac{\lambda }{2}\beta [\alpha ,\alpha ]+\beta B\right) . \end{aligned}$$

Next we formally define the functional

2.5 $$\begin{aligned} \mathcal {O}_\sigma [A,B]:=\int D\alpha \,D\beta \;\textrm{e}^{\frac{{\textrm{i}}}{\hbar }J[\alpha ,\beta ;A,B]}. \end{aligned}$$

We claim this is an observable. In fact,

$$ \delta _{\gamma ,\theta } J = \int _\Sigma \left( -\beta [{\textrm{d}}_A\gamma ,\alpha ]+\beta [\gamma ,B]-\lambda \beta [\theta ,\alpha ]-\beta {\textrm{d}}_A\theta \right) , $$

but this can be compensated by the infinitesimal transformation

$$ \alpha \leadsto \alpha -\theta -[\gamma ,\alpha ],\quad \beta \leadsto \beta -[\gamma ,\beta ], $$

in the integration variables α and β. Under the assumption that ${\mathfrak {g}}$ is unimodular, this transformation formally leaves the functional measure invariant.

As $\mathcal {O}_\sigma$ is an observable, we can consider its expectation value in the ambient four-dimensional *BF* theory. As the latter is topological, this is expected to produce an invariant of $\sigma :\Sigma \rightarrow M$. It is at this point that we should insist that σ is an embedding, otherwise the observable would not be well-defined in the BV setting and its expectation value might be singular. This expectation is made more precise in Theorem 6.5 (and more generally in Remark 6.6), where we show that indeed the expectation value is an isotopy invariant of the embedding up to a universal counterterm that has to be introduced because of anomalies. This is very similar to what happnes in Chern–Simons theory, where the Wilson loops are defined for every loop, but their expectation values are defined only for embedded loops (knots); these expectation values then have to be corrected by a universal multiple of the self-linking number in order to produce isotopy invariants.

We now turn to the problem of making some sense of the functional integral (2.5). Even if we define it perturbatively, the integral is ill-defined because of the “on-shell symmetry”

$$ \widehat{\delta }_p\alpha =-{\textrm{d}}_Ap-\lambda [\alpha ,p],\quad \widehat{\delta }_p\beta =\lambda [p,\beta ], $$

where *p* is a 0-form in $\sigma ^*\textrm{ad}P$. In fact we have

$$ \widehat{\delta }_p J = \int _\Sigma [\beta ,p](F_A-\lambda B). $$

This means that, upon using the EL Eq. (2.2) (i.e., precisely around where the functional integral in *A* and *B* will be localized), the transformation $\widehat{\delta }_p$ is a symmetry for *J*. To make the functional integral in α and β well-defined so as to make sense of (2.5), we have to impose a gauge fixing. The problem is however subtle because, as soon as we are off shell in the ambient theory, the transformation $\widehat{\delta }_p$ ceases to be a symmetry, yet we are still gauge fixing it. The right way to deal with this problem is to resort to the BV formalism and to replace (2.5) by a BV pushforward.

## The BV Formalism and the BV Pushforward

In this section, we recall the basic facts about the BV formalism [12] that we are going to use, especially the BV pushforward technique. This cannot be an exhaustive introduction, for which we refer to [30, 31] and references therein (in particular, [29, 61]), but is just a short review also aimed to fix our notations. Readers familiar with the formalism can easily skip this section.

### The classical BV formalism

The BV formalism is based on a triple $(\mathcal {F},\omega ,\mathcal {S})$, where $\mathcal {F}$ is a supermanifold, ω an odd symplectic form, and *S* an even function satisfying the Classical Master Equation (CME)

$$ (\mathcal {S},\mathcal {S})=0, $$

where $(\, , \, )$ (the BV bracket) is the odd Poisson bracket induced by ω. It is useful to introduce the hamiltonian vector field *Q* of *S*, called the BV operator,

3.1 $$\begin{aligned} \iota _Q\omega = \delta \mathcal {S}, \end{aligned}$$

where ι denotes contraction, and δ is the exterior derivative on $\mathcal {F}$. Equivalently, we have Qf=-(S,f) for every function *f* (the minus sign is also conventional).^6^ Note that $(\mathcal {S},\mathcal {S})=-Q\mathcal {S}$, so the CME is equivalent to $Q\mathcal {S}=0$. Moreover, it implies that [Q,Q]=0.

It is convenient to assume that $\mathcal {F}$ has an additional ${\mathbb {Z}}$-grading (roughly speaking, local coordinates are assigned an integer), which we call the ghost number ${{\,\textrm{gh}\,}}$ in accordance with the applications in physics. In this setting, we require ${{\,\textrm{gh}\,}}\mathcal {S}=0$ and ${{\,\textrm{gh}\,}}\omega =-1$. This implies ${{\,\textrm{gh}\,}}Q=1$, so *Q* is a differential on $C^\infty (\mathcal {F})$ (it is therefore called a cohomological vector field).

In the application to field theory, $\mathcal {F}$ is infinite-dimensional, so some care has to be taken. Typically we assume ω to be a weak symplectic form, meaning that a function has a unique hamiltonian vector field if any. Therefore, we have to assume that $\mathcal {S}$ has a hamiltonian vector field.

The passage from a classical field theory to its BV version goes through extending the space of fields *F* by adding to it the generators of the infinitesimal (on-shell) symmetries regarded as odd variables of ghost number one (the ghosts). If the symmetries are reducible (as is the case, e.g., in four-dimensional *BF* theory), ghosts-for-ghosts (of increasing ghost number and alternating parity) have also to be included. We denote this extended space of fields by $\mathcal {M}$. The BV manifold will then be $\mathcal {F}=T^*[-1]\mathcal {M}$ with its canonical symplectic form (this notation means that the momenta—the coordinates of the cotangent fiber—have opposite parity to the corresponding base coordinate and ghost number equal to the opposite of that of the base coordinate minus one). The BV action $\mathcal {S}$ has the form $\mathcal {S}=S+\cdots$, where *S* is the classical action and the dots denote terms depending on the momenta (i.e., vanishing when restricted to $\mathcal {M}$). The linear term in the momenta of the classical fields is responsible for the infinitesimal (on-shell) symmetries. Namely, if ϕ is a classical field, Qϕ is, up to terms in the momenta, the infinitesimal symmetry parametrized by the ghosts.

One notable feature of the BV formalism is that *Q* applied to the momentum of a classical field starts with the variation of *S* with respect to that field. That is, the BV formalism knows not only of the symmetries but also of the EL equations. In particular, it may combine the two and deal, in a consistent way, with symmetries that exist only on shell.

### The quantum BV formalism

The BV formalism is introduced to formalize the independence of the choice of gauge fixing. This is motivated by the following facts (see [20] and references therein, in particular:[12, 48–50, 68, 69]) which hold for a finite-dimensional, oriented, odd symplectic manifold $(\mathcal {F},\omega )$:

**(BV1):** A half-density^7^ on $\mathcal {F}$ becomes a density when restricted to a lagrangian submanifold, so it can be integrated there (if the integral converges).
**(BV2):** There is a canonical odd differential operator ▵ (in the ${\mathbb {Z}}$-version, ▵ has ghost number 1) called the BV Laplacian that acts on half-densities and satisfies $\triangle ^2=0$ and the following properties:

-       (1) $\int _\mathcal {L}\triangle \rho = 0$ for every lagrangian submanifold $\mathcal {L}$ and every half-density ρ which is integrable over $\mathcal {L}$.
-       (2) If $\triangle \rho =0$ and $\mathcal {L}_t$ is a family of lagrangian submanifolds, with ρ integrable on every $\mathcal {L}_t$, depending smoothly on a parameter *t*, then $\frac{{\textrm{d}}}{{\textrm{d}}t}\int _{\mathcal {L}_t}\rho = 0$.

#### Remark 3.1

The above can be partially understood in terms of ordinary differential geometry for $\mathcal {F}=T^*[-1]M$ with *M* an ordinary oriented manifold [68, 73]. Half-densities on $T^*[-1]M$ can be canonically identified with differential forms on *M*. Under this identification, ▵ corresponds to the exterior derivative. Stokes theorem then yields the properties of ▵ in the special case when the lagrangian submanifolds are of the form $N^*[-1]C$, where *C* is a submanifold of *M* and $N^*$ denotes the conormal bundle. The result for general lagrangian submanifolds is the discovery of Batalin and Vilkovisky [12].

Usually one prefers to work with functions instead of half-densities. To do so, one chooses^8^ a reference, nowhere vanishing half-density ρ satisfying $\triangle \rho =0$ and defines the BV Laplacian on functions by $\Delta f:=\triangle (f\rho )/\rho$. The BV Laplacian on functions clearly satisfies $\Delta ^2=0$. More surprisingly, independently of ρ, we have

$$ \Delta (fg)=(\Delta f)g + (-1)^f f\Delta g - (-1)^f (f,g), $$

where $(-1)^f=\pm 1$ denotes the parity of *f* and $(\, , \, )$ is the BV bracket. In the case of the exponential of an even function *f*, one has

3.2 $$\begin{aligned} \Delta \textrm{e}^f= \left( \Delta f-\frac{1}{2}(f,f)\right) \textrm{e}^f. \end{aligned}$$

### BV functional integrals

The application to physics is the following setting. One starts with a classical action *S* on a space *F* of classical fields. The goal is to make sense of the functional integral $\int _F\textrm{e}^{\frac{{\textrm{i}}}{\hbar }S}$. If the action has symmetries, the critical points are degenerate, so the saddle point approximation cannot be used. One then introduces the space $\mathcal {G}$ of generators of the infinitesimal symmetries and considers instead the integral $\int _{F\times \mathcal {G}[1]}\textrm{e}^{\frac{{\textrm{i}}}{\hbar }S}$. Since we have made the generators odd via the shift in $\mathcal {G}[1]$ (they are now the ghosts), we have to compute a berezinian integral, which vanishes because the classical action does not depend on the ghosts. Therefore, the integral is of the form infinity times zero—undetermined but already better than just infinity.

The next step is to consider the odd symplectic manifold $\mathcal {F}:=T^*[-1](F\times \mathcal {G}[1])$, in which $\mathcal {L}_0:=F\times \mathcal {G}[1]$ sits as a lagrangian submanifold, and to extend the classical action to a functional $\mathcal {S}$ such that

1. $$\mathcal {S}|_{\mathcal {L}_0}=S$$, and
2. $$\Delta \textrm{e}^{\frac{{\textrm{i}}}{\hbar }\mathcal {S}}=0$$.

We now consider a family $\mathcal {L}_t$ of lagrangian submanifolds deforming $\mathcal {L}_0$ such that the critical points of $\mathcal {S}$ on $\mathcal {L}_t$ are nondegenerate for t≠0. We then replace the ill-defined integral $\int _{\mathcal {L}_0}\textrm{e}^{\frac{{\textrm{i}}}{\hbar }\mathcal {S}}$ with $\int _{\mathcal {L}_t}\textrm{e}^{\frac{{\textrm{i}}}{\hbar }\mathcal {S}}$, t≠0, where the saddle-point approximation now makes sense. By the BV property (BV2.2), it does not matter which $\mathcal {L}_t$, t≠0, we choose. Such an $\mathcal {L}_t$ is called a gauge-fixing Lagrangian.^9^

The main problem in the infinite-dimensional case is that the BV Laplacian does not make sense as it is: it can be properly defined only after a regularization is introduced.

However, by (3.2), the condition $\Delta \textrm{e}^{\frac{{\textrm{i}}}{\hbar }\mathcal {S}}=0$ is equivalent to the Quantum Master Equation (QME)

$$ \frac{1}{2}(\mathcal {S},\mathcal {S})-{\textrm{i}}\hbar \Delta \mathcal {S}=0. $$

Here we see that the ill-defined term $\Delta \mathcal {S}$ occurs at the first order in ħ, so it can be ignored as a first approximation. That is, we are only going to assume that $\mathcal {S}$ satisfies the CME $(\mathcal {S},\mathcal {S})=0$. The functional integral is then defined in terms of Feynman diagrams, and, a posteriori, we check that it is indeed invariant under deformations of the gauge-fixing Lagrangian. (More correctly, one should regularize the theory while defining the Feynman diagrams and consistently check the QME in the regularized version; see [58, 59] and especially [35, 36]).

With this caveat, we can now make sense of the partition function $Z:=\int _{\mathcal {L}_t}\textrm{e}^{\frac{{\textrm{i}}}{\hbar }S}\rho$, where ρ is the reference half-density. The next goal is to consider functionals $\mathcal {O}$ on $\mathcal {F}$ for which the expectation value

$$ \langle \mathcal {O}\rangle :=\frac{1}{Z}\int _{\mathcal {L}_t}\textrm{e}^{\frac{{\textrm{i}}}{\hbar }S}\mathcal {O}\rho $$

is also invariant under deformations of the gauge-fixing Lagrangian. By the BV property (BV2.2), this happens if $\Delta \left( \textrm{e}^{\frac{{\textrm{i}}}{\hbar }\mathcal {S}}\mathcal {O}\right) =0$, that is, assuming the QME, if

$$ Q\mathcal {O}-{\textrm{i}}\hbar \Delta \mathcal {O}=0, $$

where, as above, $Q=-(\mathcal {S},\ )$. We call such a functional a quantum BV observable.

#### Remark 3.2

*(Quantum BV cohomology)*. It is convenient to introduce the operator $\Omega :-Q - {\textrm{i}}\hbar \Delta$, which is a coboundary operator thanks to the QME. BV observables are by definition Ω-closed. On the other hand, by property (BV2.1), the expectation value of an Ω-exact functional vanishes. As a consequence, a BV theory, with a choice of a lagrangian submanifold up to deformations, is a linear functional on the cohomology $H_\Omega$ of Ω (to be precise, we should only consider classes with a representative for which the functional integral makes sense.).

Again, the ill-defined term $\Delta \mathcal {O}$ occurs at the first order in ħ, so we will at first ignore it. We therefore define a classical BV observable as a functional $\mathcal {O}$ on $\mathcal {F}$ satisfying $Q\mathcal {O}=0$.

It follows that $\mathcal {O}|_{\mathcal {L}_0}$ is a functional on *F* that is invariant under the infinitesimal symmetries. Usually one has to work the other way around, starting with a classical invariant functional and looking for an extension to a classical BV observable. Our goal will be to construct a classical BV observable $\mathcal {O}_\sigma$ for *BF* theory with cosmological term which, on $\mathcal {L}_0$, reduces to (2.5).

### The BV pushforward

In this section we describe a partial BV integration (BV pushforward) which is needed for the rest of the paper; see [27, 29, 33, 35, 36, 55, 58, 59].

We assume that our odd symplectic manifold $(\mathcal {F},\omega )$ is the product^10^ of two odd symplectic manifolds $(\mathcal {F}_1,\omega _1)$ and $(\mathcal {F}_2,\omega _2)$: $\mathcal {F}=\mathcal {F}_1\times \mathcal {F}_2$, $\omega =\pi _1^*\omega _1+\pi _2^*\omega _2$, with $\pi _i$ the canonical projection $\mathcal {F}\rightarrow \mathcal {F}_i$. The canonical BV Laplacian ▵ is then also a sum $\triangle =\triangle _1+\triangle _2$. If ρ is a half-density on $\mathcal {F}$ and $\mathcal {L}_2$ is a lagrangian submanifold of $\mathcal {F}_2$, then $\int _{\mathcal {L}_2}\rho$ is a half-density on $\mathcal {F}_1$ (assuming that the integral converges). Moreover, we have:

**BVpf1:** $$\triangle _1\int _{\mathcal {L}_2}\rho =\int _{\mathcal {L}_2}\triangle \rho $$, and
**BVpf2:** $\frac{{\textrm{d}}}{{\textrm{d}}t}\int _{\mathcal {L}_{2,t}}\rho$ is $\triangle _1$-exact if ρ is ▵-closed.

This means that the partial BV integration is a well-defined^11^ operation in the cohomologies of the BV Laplacians (as long as we choose a deformation class of lagrangian submanifolds of $\mathcal {F}_2$). This operation is called the BV pushforward.

We now fix a reference, ▵-closed, nowhere vanishing half-density ρ on $\mathcal {F}$. It follows that $\rho _1:=\int _{\mathcal {L}_2}\rho$ is also ▵-closed and nowhere vanishing. If $\mathcal {S}$ is a solution of the QME master equation, we define $\mathcal {S}_\text {eff}$ via

$$ \textrm{e}^{\frac{{\textrm{i}}}{\hbar }\mathcal {S}_\text {eff}}\rho _1= \int _{\mathcal {L}_2}\textrm{e}^{\frac{{\textrm{i}}}{\hbar }\mathcal {S}}\rho . $$

The new BV action $\mathcal {S}_\text {eff}$ (called the effective action) solves the QME on $\mathcal {F}_1$ and is defined up to quantum BV transformations (i.e., $\textrm{e}^{\frac{{\textrm{i}}}{\hbar }\mathcal {S}_\text {eff}}$ is defined up to $\Delta _1$-exact terms). We call $(\mathcal {F},\omega ,\mathcal {S})$ the parent theory and $(\mathcal {F}_1,\omega _1,\mathcal {S}_\text {eff})$ the effective theory (we borrow the terminology from [11]).

#### Remark 3.3

*(Pushforward of observables)*. If $\mathcal {O}$ is a quantum BV observable for the parent theory (i.e., $\Delta \left( \textrm{e}^{\frac{{\textrm{i}}}{\hbar }\mathcal {S}}\mathcal {O}\right) =0$), we define the corresponding quantum BV observable $\mathcal {O}_1$ for the effective theory via

3.3 $$\begin{aligned} \textrm{e}^{\frac{{\textrm{i}}}{\hbar }\mathcal {S}_\text {eff}}\mathcal {O}_1\rho _1= \int _{\mathcal {L}_2}\textrm{e}^{\frac{{\textrm{i}}}{\hbar }\mathcal {S}}\mathcal {O}\rho . \end{aligned}$$

This way, one can construct quantum BV observables for the the effective theory starting from quantum BV observables for the parent theory. We will use this procedure in Sect.  7.

#### BV quasiisomorphism

More generally, we can think of (3.3) as a map from functions on $\mathcal {F}$ to functions on $\mathcal {F}_1$. By property (BVpf1), we see that this is actually a chain map with respect to the coboundary operators $\Omega =Q+{\textrm{i}}\hbar \Delta$ and $\Omega _1=Q_1+{\textrm{i}}\hbar \Delta _1$, see Remark 3.2. As such, it induces a map in the corresponding cohomologies $H_\Omega$ and $H_{\Omega _1}$.

If this induced map is an isomorphism, one says that the chain map between functionals is a quasiisomorphism. This is the case under some assumptions which are (formally) satisfied by all examples in this note. One simple setting is when $\mathcal {S}$ is a perturbation of abelian *BF* theory and the gauge-fixing Lagrangian comes from a chain homotopy operator. Strictly speaking, this is a theorem [58, 59] (see also [29, 40] and the forthcoming [28] for a more general discussion) in the finite-dimensional case—in the infinite-dimensional one, the BV Laplacian is not even properly defined—but we formally extend it to field theory. Note that the semiclassical part of this (i.e., the restriction to only trees in the expansion in Feynman diagrams) yields back the homotopy transfer by Merkulov [57] and Kontsevich–Soibelman [52].

### Construction of observables

Another way to obtain quantum BV observables for a BV theory is the following [33]. Suppose that $\mathcal {S}=\pi _1^*\mathcal {S}_{(1)}+\mathcal {S}_{(2)}$ with $\mathcal {S}_{(1)}$ solving the QME on $\mathcal {F}_1$ (we still also assume that $\mathcal {S}$ solves the QME on $\mathcal {F}$). We also assume that $\rho =\rho _1\rho _2$, with $\rho _1$ the induced reference half-density on $\mathcal {F}_1$ and $\rho _2$ a reference, ▵-closed, nowhere vanishing half-density on $\mathcal {F}_2$. Then

$$ \textrm{e}^{\frac{{\textrm{i}}}{\hbar }\mathcal {S}_\text {eff}}\rho _1=\textrm{e}^{\frac{{\textrm{i}}}{\hbar }\mathcal {S}_{(1)}}\rho _1\int _{\mathcal {L}_2}\textrm{e}^{\frac{{\textrm{i}}}{\hbar }\mathcal {S}_{(2)}}\rho _2, $$

and we get the quantum BV observable

3.4 $$\begin{aligned} \mathcal {O}_1 = \int _{\mathcal {L}_2}\textrm{e}^{\frac{{\textrm{i}}}{\hbar }\mathcal {S}_{(2)}}\rho _2 \end{aligned}$$

for the BV theory $(\mathcal {F}_1,\omega _1,\mathcal {S}_{(1)})$ (note that this is not the effective theory). We will use this procedure in Sect.  4.

### Residual fields

A special case of the BV pushforward construction is when $\mathcal {F}_1$ is finite-dimensional. In this case, following [29], we call it the space of residual fields.

There can be different choices, also with different dimensions, of spaces of residual fields. Assuming they are all quasiisomorphic to each other, we consider them as fully equivalent. Usually there is a minimal choice, but in some cases (e.g., when one wants to glue theories along a boundary [29]) it is not the most convenient.

More generally, we think of $\mathcal {F}_2$ as a product $\mathcal {F}_\text {res}\times \widehat{\mathcal {F}}_2$, where $\mathcal {F}_\text {res}$ is finite-dimensional. In this case, when we speak of a BV pushforward along $\mathcal {F}_2$, we actually mean a BV pushforward along $\widehat{\mathcal {F}}_2$ yielding an effective theory on $\mathcal {F}_1\times \mathcal {F}_\text {res}$.

In particular, the construction of Sect.  3.5 yields a quantum BV observable $\mathcal {O}_1$, see Eq. (3.4), for the BV theory $(\mathcal {F}_1,\omega _1,\mathcal {S}_{(1)})$ parametrized over $(\mathcal {F}_\text {res},\omega _\text {res},\mathcal {S}_\text {res})$. An appropriate integration over the latter space may sometimes be performed (one notable example is described in [14]).

## BV Surface Observables for *BF* Theory

*BF* theory is an example of an AKSZ [1] theory, so its BV version is easily constructed. We give the final answer, as it is immediate to verify that the BV action we write extends the classical action (2.1) and satisfies the master equation. We first introduce the “superfields”

4.1 $$\begin{aligned} \begin{aligned} \mathcal {A}&= c + A + B^+ + \tau ^+ + \phi ^+\!,\\ \mathcal {B}&= \phi + \tau + B + A^+ + c^+\!. \end{aligned} \end{aligned}$$

In each superfield, the fields (with the exception of *A*, which is a connection 1-form) are $\textrm{ad}$-valued differential forms ordered by increasing form degree (with *c* and ϕ 0-forms and with $\phi ^+$ and $c^+$ 4-forms). They are assigned ghost numbers in decreasing order starting with *c* of ghost number 1 and ϕ of ghost number 2 (in other words, a summand in $\mathcal {A}$ has ghost number equal to 1 minus its form degree, and a summand in $\mathcal {B}$ has ghost number equal to 2 minus its form degree). Here *A* and *B* are the classical fields already appearing in (2.1), whereas *c* and τ are the ghosts corresponding to the infinitesimal symmetries (2.3). We have to introduce the ghost-for-ghost ϕ in order to have the infinitesimal action injective also on-shell. The fields with a + superscripts are the momenta. More precisely, we have the BV symplectic form

$$ \omega :=\int _M \delta \mathcal {B}\,\delta \mathcal {A}, $$

where $\int _M$ selects the summands of form degree equal to 4 (therefore, of ghost number -1). The BV action is

4.2 $$\begin{aligned} \mathcal {S}:=\int _{M}\left( \mathcal {B}F_\mathcal {A}-\dfrac{\lambda }{2}\mathcal {B}\mathcal {B}\right) , \end{aligned}$$

where we use the same convention as for the BV symplectic form and $F_\mathcal {A}:={\textrm{d}}\mathcal {A}+\frac{1}{2}[\mathcal {A},\mathcal {A}]$ is the “curvature of the superconnection $\mathcal {A}$.”

The hamiltonian vector field *Q* of $\mathcal {S}$, see (3.1), can be easily computed. We should only be careful with signs.

### Remark 4.1

*(Sign conventions)*. Throughout the paper, we use the total degree, i.e., the sum of form and ghost degree, to determine the parity of an object and the consequent sign rules. According to this convention, each summand in $\mathcal {A}$ is odd and each summand in $\mathcal {B}$ is even. Moreover, the operators ${\textrm{d}}_A$ and *Q* are odd, but the contraction $\iota _Q$ is even. Finally, we need the odd operator ${\textrm{d}}_\mathcal {A}$, which is locally defined as ${\textrm{d}}+[\mathcal {A},\ ]$.

Following these conventions, we have^12^

4.3a $$\begin{aligned} Q\mathcal {A}&= F_\mathcal {A}-\lambda \mathcal {B},\end{aligned}$$

4.3b $$\begin{aligned} Q\mathcal {B}&= {\textrm{d}}_\mathcal {A}\mathcal {B}. \end{aligned}$$

One can easily see that $(\mathcal {S},\mathcal {S})=-Q\mathcal {S}=0$. Moreover, formally we also have $\Delta \mathcal {S}=0$ if we assume the Lie algebra to be unimodular.

We denote by $\mathcal {F}_M$ the BV space of fields. If $A_0$ is a reference connection, we have $\mathcal {F}_M=A_0+\Omega ^\bullet (M,\textrm{ad}P)[1]\oplus \Omega ^\bullet (M,\textrm{ad}P)[2]$.^13^

To get the BV action corresponding to *J* in (2.4), we follow a similar strategy as above. First we introduce the superfields

$$ \begin{aligned} a&= \psi + \alpha + \beta ^+,\\ b&= \beta + \alpha ^+ + \psi ^+, \end{aligned} $$

following the same conventions for form degree and ghost number (just note that ψ has ghost number 1, whereas β has ghost number 0). All the summands here are sections of the $\textrm{ad}$-bundle pulled back to Σ. We denote this space of fields by $\mathcal {F}_\Sigma =\Omega ^\bullet (\Sigma ,\textrm{ad}P)[1]\oplus \Omega ^\bullet (\Sigma ,\textrm{ad}P)$.

We extend the BV symplectic form ω by adding a term $\omega _\Sigma :=\int _\Sigma \delta b\,\delta a$. Finally, we consider the BV action

$$ \mathcal {J}[a,b;\mathcal {A},\mathcal {B}] :=\int _\Sigma \left( b\,{\textrm{d}}_\mathcal {A}a+\frac{\lambda }{2} b[a,a]+b \mathcal {B}\right) $$

and define

$$ \widehat{\mathcal {S}}:=\mathcal {S}+\mathcal {J}. $$

We denote by $\widehat{Q}$ its hamiltonian vector field.

### Lemma 4.2

$\widehat{\mathcal {S}}$ satisfies the classical master equation $(\widehat{\mathcal {S}},\widehat{\mathcal {S}})=-\widehat{Q}\widehat{\mathcal {S}}=0$.

### Proof

We denote by *R* the hamiltonian vector field of $\mathcal {J}$. An easy computation yields

$$ \begin{aligned} Ra&= {\textrm{d}}_\mathcal {A}a+\frac{\lambda }{2}[a,a]+\mathcal {B},\\ Rb&= {\textrm{d}}_\mathcal {A}b+\lambda [a,b],\\ R\mathcal {A}&= b\,\delta _\Sigma ,\\ R\mathcal {B}&= [a,b]\,\delta _\Sigma , \end{aligned} $$

where $\delta _\Sigma$ is the delta 2-form localized on Σ. We have $\widehat{Q}=Q+R$ and

$$ \widehat{Q}\widehat{\mathcal {S}}=(Q+R)(\mathcal {S}+\mathcal {J})=Q\mathcal {S}+Q\mathcal {J}+R\mathcal {S}+R\mathcal {J}. $$

We already mentioned that $Q\mathcal {S}=0$. An explicit computation shows

$$ Q\mathcal {J}=R\mathcal {S}=\int _\Sigma \left( b[F_\mathcal {A},a]-\lambda b[\mathcal {B},a]+b\,{\textrm{d}}_\mathcal {A}\mathcal {B}\right) . $$

Finally, another explicit computation yields $R\mathcal {J}=-2Q\mathcal {J}$. □

The above proof is a bit unsatisfactory, as some terms are actually singular. In fact, in the computation of $R\mathcal {S}$, we also get the terms

$$ \int _\Sigma (b[b,a]+b[a,b])\delta _\Sigma . $$

The delta 2-form $\delta _\Sigma$ evaluated on Σ is singular. It should be regularized as a Poincaré dual of Σ. On the other hand, the term in the round bracket clearly vanishes, so formally this term is zero. At this level it is however irrelevant to make this argument more rigorous, since we will check a posteriori, see Proposition 5.3, that the BV pushforward produces a quantum BV observable for the ambient *BF* theory.

Remaining at the same heuristic level, we can however also conclude that $\Delta \mathcal {J}=0$ under the assumptions that the Lie algebra is unimodular and that $\sigma :\Sigma \rightarrow M$ is an embedding. This also implies that $\Delta \widehat{\mathcal {S}}=0$, so $\widehat{\mathcal {S}}$ satisfies the quantum master equation.

As a consequence, a BV pushforward on the fields *a* and *b* as in (3.4) formally yields a quantum BV observable $\mathcal {O}_\sigma$ for *BF* theory, replacing the naive definition (2.5). More precisely,

$$ \mathcal {O}_\sigma :=\int _{\mathcal {L}_2}\textrm{e}^{\frac{{\textrm{i}}}{\hbar }\mathcal {J}}\rho _2. $$

We will avoid the issues of the heuristic computations arising in this section by using the perturbative formulation of the observable $\mathcal {O}_\sigma$ in Sect.  5.

### Isotopy invariance

We conclude this section with a heuristic proof that the expectation value of $\mathcal {O}_\sigma$ is a knot invariant (see Theorem 6.5 and Remark 6.6 for details on the actual proof). This has to be expected as we are dealing with a topological field theory. It is however instructive to view a direct argument using the BV formalism.

Let $\phi :M\times [0,1]\rightarrow M$ be an isotopy relating two embeddings $\sigma _0$ and $\sigma _1$ of Σ into *M*. Let $\xi _t$ be the corresponding vector field at t∈[0,1].

In case the principal bundle *P* is trivial and we regard all the fields, including the connection *A*, as ${\mathfrak {g}}$-valued differential forms (i.e., we work around the trivial connection), we define

$$ \mathcal {U}_t :=\int _\Sigma \left( b\,[\iota _{\xi _t}\mathcal {A}, a]+b\, \iota _{\xi _t}\mathcal {B}\right) . $$

A simple computation, by the same heuristics as before, shows that

$$ \widehat{Q}\mathcal {U}_t = \int _\Sigma \left( b\,[\textsf{L}_{\xi _t}\mathcal {A}, a]+b\, \textsf{L}_{\xi _t}\mathcal {B}\right) = \frac{{\textrm{d}}}{{\textrm{d}}t} \mathcal {J}_t, $$

where $\textsf{L}$ denotes the Lie derivative. Since, also formally, $\Delta \mathcal {U}_t=0$, we get

$$ \frac{{\textrm{d}}}{{\textrm{d}}t} \mathcal {O}_\sigma = \Omega \int _{\mathcal {L}_2}\textrm{e}^{\frac{{\textrm{i}}}{\hbar }\mathcal {J}}\mathcal {U}_t\rho _2, $$

so the the *t*-derivative of the expectation value of $\mathcal {O}_\sigma$ in *BF* theory vanishes.

If the principal bundle *P* is not trivial, we lift the vector fields $\xi _t$ to *P* by a reference connection $A_0$. In this case, we have

$$ \frac{{\textrm{d}}}{{\textrm{d}}t} \mathcal {J}_t = \int _\Sigma \left( b\,[\iota _\xi F_{A_0}+\textsf{L}^{A_0}_{\xi _t}\widehat{\mathcal {A}}, a]+b\, \textsf{L}^{A_0}_{\xi _t}\mathcal {B}\right) , $$

where $\widehat{\mathcal {A}}=\mathcal {A}-A_0$ and $\textsf{L}^{A_0}_{\xi _t}=[\iota _\xi ,{\textrm{d}}_{A_0}]$. We now define

$$ \mathcal {U}_t :=\int _\Sigma \left( b\,[\iota _{\xi _t}\widehat{\mathcal {A}}, a]+b\, \iota _{\xi _t}\mathcal {B}\right) $$

and verify that $\widehat{Q}\mathcal {U}_t=\frac{{\textrm{d}}}{{\textrm{d}}t} \mathcal {J}_t$. The rest of the argument is as in the trivial case.

### Topological deformations and other observables

The setting presented so far can be modified to a certain extent. This will be useful in connection with Yang–Mills theory (see Sect.  7).

#### Topological deformations

The functional

$$ \mathcal {S}_\text {top}^\kappa :=\frac{\kappa }{2}\int _MF_\mathcal {A}F_\mathcal {A}, $$

where κ is a parameter, satisfies $\delta \mathcal {S}_\text {top}^\kappa =0$ (for *M* closed), so it can be used to deform the *BF* action (4.2) to

$$ \mathcal {S}^\kappa :=\mathcal {S}+ \mathcal {S}_\text {top}^\kappa , $$

which still satisfies the classical (and formally the quantum) master equation. Also note that the hamiltonian vector field of $\mathcal {S}^\kappa$ is the same as that of $\mathcal {S}$. As a consequence,

$$ \widehat{\mathcal {S}}^\kappa :=\mathcal {S}^\kappa +\mathcal {J}$$

still satisfies the classical (and formally the quantum) master equation for any κ. Therefore, the BV pushforward of $\textrm{e}^{\frac{{\textrm{i}}}{\hbar }\mathcal {J}}$ defines a surface observable for $\mathcal {S}^\kappa$ with any κ.

#### Other surface observables

Consider the transformation

$$ \mathcal {A}\mapsto \mathcal {A},\qquad \mathcal {B}\mapsto \mathcal {B}+\gamma F_\mathcal {A}, $$

for some parameter γ. This is a quantum BV transformation (i.e., a symplectomorphisms that, formally, does not change the reference “Lebesgue” half-density). The action is transformed accordingly, and a simple computation yields

$$ \mathcal {S}^\kappa \rightarrow (1-\lambda \gamma )\mathcal {S}' + \mathcal {S}_\text {top}^{\kappa +\gamma -\frac{\lambda \gamma ^2}{2}} $$

with

$$ \mathcal {S}':=\int _{M}\left( \mathcal {B}F_\mathcal {A}-\dfrac{\lambda '}{2}\mathcal {B}\mathcal {B}\right) $$

and

$$ \lambda '=\frac{\lambda }{1-\gamma \lambda }. $$

It follows that $$(1-\lambda \gamma )(\mathcal {S}'+\mathcal {J}')$$ and $$(1-\lambda \gamma )(\mathcal {S}'+\mathcal {J}')+ \mathcal {S}_\text {top}^{\kappa +\gamma -\frac{\lambda \gamma ^2}{2}}$$, with $$\mathcal {J}'$$ obtained from $$\mathcal {J}$$ replacing λ with $\lambda^{\prime }$, still satisfy the classical (and formally the quantum) master equation.

We can now revert the transformation getting back $\mathcal {S}^\kappa$. This transforms $(1-\lambda \gamma )\mathcal {J}'$ into

4.4 $$\begin{aligned} \mathcal {J}_\gamma :=(1-\lambda \gamma )\int _\Sigma \left( b\,{\textrm{d}}_\mathcal {A}a+\frac{\lambda '}{2} b[a,a]+b \mathcal {B}-\gamma b F_\mathcal {A}\right) . \end{aligned}$$

In conclusion,

$$ \widehat{\mathcal {S}}^\kappa _\gamma :=\mathcal {S}^\kappa +\mathcal {J}_\gamma $$

still satisfies the classical (and formally the quantum) master equation for any κ and γ. Therefore, for every γ, the BV pushforward of $\textrm{e}^{\frac{{\textrm{i}}}{\hbar }\mathcal {J}_\gamma }$ defines a surface observable for $\mathcal {S}^\kappa$ with any κ.

## Perturbative Expansion

To start the perturbative expansion, we have to fix a critical point $(A_0,B_0)$ for the ambient theory and then pick a critical point $(\alpha _0,\beta _0)$ for the theory on Σ evaluated at $(A_0,B_0)$.^14^

We prefer however to consider also λ as a perturbative parameter. Therefore, we assume $F_{A_0}=0$ and ${\textrm{d}}_{A_0}B_0=0$. We can then rewrite the action on Σ at $(A_0,B_0)$ as

$$ \mathcal {J}[a,b;A_0,B_0] :=\int _\Sigma \left( b\,{\textrm{d}}_{A_0} a+ b B_0\right) + O(\lambda ). $$

A critical point for the classical fields of the theory on Σ is now a solution of

$$ {\textrm{d}}_{A_0} \beta _0 = 0\quad \text {and}\quad {\textrm{d}}_{A_0} \alpha _0+ B_0=0, $$

and we complete it to a critical point for the BV theory by setting all the other BV fields to zero. We write

5.1 $$\begin{aligned} \begin{aligned} a&= \alpha _0 + \widehat{a},\\ b&= \beta _0 + \widehat{b},\\ \mathcal {A}&= A_0 + \widehat{\mathcal {A}},\\ \mathcal {B}&= B_0 + \widehat{\mathcal {B}}, \end{aligned} \end{aligned}$$

where the hatted fields are the perturbations. The spaces of fields were defined in Section 4. Accordingly, we have

$$\begin{aligned} \widehat{a}&\in \mathcal {F}_\Sigma =\Omega ^\bullet (\Sigma ,\textrm{ad}P)[1],&\widehat{b}&\in \Omega ^\bullet (\Sigma ,\textrm{ad}P), \\ \widehat{\mathcal {A}}&\in \Omega ^\bullet (M,\textrm{ad}P)[1],&\widehat{\mathcal {B}}&\in \Omega ^\bullet (M)[2]. \end{aligned}$$

With this decomposition, we get

$$ \mathcal {J}[a,b;\mathcal {A},\mathcal {B}] = \int _\Sigma \widehat{b}\,{\textrm{d}}_{A_0} \widehat{a} + \text {perturbations}. $$

The gauge-fixing Lagrangian $\mathcal {L}_2$, and the choice of residual fields, have then to be chosen in such way that on $\mathcal {L}_2$ the operator ${\textrm{d}}_{A_0}$ can be inverted.

### Remark 5.1

*(Result of the perturbative expansion)*. The gauge-fixed BV integral will ultimately yield a “function" $\mathcal {O}_\sigma$ of the background fields ($\alpha _0,\beta _0)$ and of the ambient fields $(\mathcal {A},\mathcal {B})$. To be more precise, we will have to split the hatted fields $(\widehat{a},\widehat{b})$ into residual fields $(\underline{a},\underline{b})$ and fluctuations $(\widetilde{a},\widetilde{b})$,

5.2 $$\begin{aligned} \widehat{a} = \underline{a} + \widetilde{a},\qquad \widehat{b} = \underline{b} + \widetilde{b}, \end{aligned}$$

where the residual fields are in the cohomology of ${\textrm{d}}_{A_0}$, with a choice of embedding into the space of hatted fields and a choice of complement, which in turn defines the space of fields of a tilde. The result of the gauge-fixed BV integral over the fluctuations $(\widetilde{a},\widetilde{b})$ will then yield $\mathcal {O}_\sigma$ as a half-density on the space of residual fields $(\underline{a},\underline{b})$, parametrized by the background fields ($\alpha _0,\beta _0)$ and the ambient fields $(\mathcal {A},\mathcal {B})$. The starting point of the perturbative computation is the computation of the Berezinian of the quadratic form: this yields the analytic torsion for the complex with differential ${\textrm{d}}_{A_0}$, which is indeed a half-density over the space of residual fields $(\underline{a},\underline{b})$. The full perturbative expansion multiplies this torsion by an expansion in Feynman diagrams. The procedure is the following (in brackets we point at the related sections for the trivial case):

1. Get a BV symplectic splitting of the spaces of fields into residual fields and fluctuations, and choose a BV gauge fixing, i.e., a lagrangian submanifold in the BV space of fluctuations (Section 5.1).
2. Compute the propagator for the fluctuations with the chosen gauge fixing, i.e., invert the (now nondegenerate) bilinear form appearing as the term in the action that is quadratic in the fluctuations (Sects.  5.1.1 and 5.1.2).
3. Interpret the functional integral as a formal expansion around the quadratic term. This leads to Feynman diagrams, whose edges are the propagators and whose vertices correspond to the other terms in the action. Next regularize the Feynman diagrams. With our choices, this regularization will amount to working with the Axelrod–Singer compatification [6, 7] of configuration spaces (Sect.  5.1.3). In this regularization, the Feynman diagrams will correspond to integrals of smooth forms on compact manifolds (with boundary), so no divergences will arise. On the other hand, Stokes theorem will be available to prove properties of the Feynman diagrams (Proposition 6 and Sect.  6.1.2).

### The perturbative expansion in the trivial case

For simplicity, we will only consider the case when the principal bundle *P* is trivial and will work around $A_0=0$ and $B_0=0$. In this case ${\textrm{d}}_{A_0}$ is just the exterior derivative ${\textrm{d}}$ (tensor the identity on ${\mathfrak {g}}$). We fix a Riemannian metric on Σ and consider the related Hodge decomposition^15^

$$ \Omega ^\bullet (\Sigma ) = {\textrm{Harm}}^\bullet (\Sigma ) \perp \hspace{-11.49994pt}\bigcirc \ {\textrm{d}}\Omega ^{\bullet -1}(\Sigma ) \perp \hspace{-11.49994pt}\bigcirc \ {\textrm{d}}^* \Omega ^{\bullet +1}(\Sigma ), $$

where ${\textrm{d}}^*$ is the Hodge codifferential and ${\textrm{Harm}}$ denotes the space of harmonic forms. According to Sect.  3.6, we rewrite the BV space of fields $\mathcal {F}_\Sigma =(\Omega ^\bullet (\Sigma )[1]\oplus \Omega ^\bullet (\Sigma ))\otimes {\mathfrak {g}}$ as

$$ \mathcal {F}_\Sigma = \mathcal {F}_{\Sigma ,\text {res}} \times \widehat{\mathcal {F}}_\Sigma $$

with $\mathcal {F}_{\Sigma ,\text {res}}=({\textrm{Harm}}^\bullet (\Sigma )[1]\oplus {\textrm{Harm}}^\bullet (\Sigma ))\otimes {\mathfrak {g}}$ and $\widehat{\mathcal {F}}_\Sigma$ the space of exact and coexact ${\mathfrak {g}}$-valued forms. Inside $\widehat{\mathcal {F}}_\Sigma$ we choose the Lagrangian submanifold

$$ \mathcal {L}_2 :=({{\,\textrm{im}\,}}{\textrm{d}}^*)\otimes {\mathfrak {g}}. $$

#### The propagator

To construct the propagator, we focus first on the abelian theory and rewrite $\int _\Sigma \widehat{b}\,{\textrm{d}}\widehat{a}$ as $\langle \widehat{b},*^{-1}{\textrm{d}}\widehat{a}\rangle$, where $\langle \eta ,\zeta \rangle =\int _\Sigma \eta *\!\zeta$ is the Hodge pairing of forms. The propagator is then the inverse of $*^{-1}{\textrm{d}}$ on ${\textrm{d}}^* \Omega ^{\bullet }(\Sigma )$. We look for the inverse *L* of $*{\textrm{d}}$ which is related to the propagator by a sign operator. We claim that

$$ L = *{\textrm{d}}G, $$

where *G* is the inverse of the Laplace operator ${\textrm{d}}^*{\textrm{d}}+{\textrm{d}}{\textrm{d}}^*$, which is invertible in the orthogonal complement of ${\textrm{Harm}}^\bullet (\Sigma )$. In fact, we have $*{\textrm{d}}L= {\textrm{d}}^*{\textrm{d}}G = G {\textrm{d}}^*{\textrm{d}}$. Therefore, for any γ,

$$ *{\textrm{d}}L{\textrm{d}}^*\gamma =G {\textrm{d}}^*{\textrm{d}}{\textrm{d}}^*\gamma =G({\textrm{d}}^*{\textrm{d}}+{\textrm{d}}{\textrm{d}}^*){\textrm{d}}^*\gamma ={\textrm{d}}^*\gamma , $$

so $*{\textrm{d}}L$ acts as the identity on the image of ${\textrm{d}}^*$. This is the propagator as considered, in Chern–Simons theory, by Axelrod and Singer [6, 7].

It is convenient to extend *G* to the de Rham complex $\Omega ^\bullet (\Sigma )$ defining it to be zero on ${\textrm{Harm}}^\bullet (\Sigma )$. Next we define

5.3 $$\begin{aligned} K :={\textrm{d}}^* G. \end{aligned}$$

It is clear that *L* is, up to a sign operator, the restriction to ${\textrm{d}}^* \Omega ^{\bullet }(\Sigma )$ of K∗. Moreover, we have

5.4 $$\begin{aligned} {\textrm{d}}K + K {\textrm{d}}= \textrm{id}- \pi _{{\textrm{Harm}}}, \end{aligned}$$

where $\pi _{{\textrm{Harm}}}$ is the orthogonal projection to ${\textrm{Harm}}^\bullet (\Sigma )$. In other words, *K* is a parametrix for ${\textrm{d}}$. Using another terminology, $(K,\iota ,\pi _{{\textrm{Harm}}})$, where ι is the inclusion map of ${\textrm{Harm}}^\bullet (\Sigma )$, is a deformation retract for the de Rham complex $(\Omega ^\bullet (\Sigma ),{\textrm{d}})$. This is at the basis of the homotopy transfer, which will correspond to the tree-level Feynman diagrams in the perturbative expansion.

#### The soft propagator

A more general and flexible definition of propagator, which we will call a soft propagator, is any operator *K* satisfying (5.4).^16^ A propagator in this sense can always be constructed by soft methods as in [17] (see also [19]). Namely, one can write

$$ K\alpha = -\pi _{2*}(\eta \,\pi _1^*\alpha ), \qquad \alpha \in \Omega ^\bullet (\Sigma ), $$

where $\pi _1$ and $\pi _2$ are the two projections $\Sigma \times \Sigma \rightarrow \Sigma$ and the lower ∗ denotes integration along the fiber (pushforward). Here η is the (soft) propagator kernel as a 1-form^17^ on $\Sigma \times \Sigma$ with a singularity on the diagonal $\triangle _\Sigma$. It is then convenient to remove the diagonal and to consider the configuration space $C_2^0(\Sigma ):=\Sigma \times \Sigma \setminus \triangle _\Sigma$, on which η is smooth.

Even better, we consider the compactification $C_2(\Sigma )$ obtained as the closure of $C_2^0(\Sigma )$ inside $\Sigma \times \Sigma \times {\textrm{Bl}}(\triangle _\Sigma )$, where ${\textrm{Bl}}(\triangle _\Sigma )$ is the differential-geometric blowup obtained by replacing $\triangle _\Sigma$ inside $\Sigma \times \Sigma$ with its unit normal bundle.^18^ We keep denoting by $\pi _1$ and $\pi _2$ the two projections to Σ. Now, $\pi _2^{-1}(x)$ is a compact manifold for every *x* in Σ, so the the pushforward $\pi _{2*}$ is integration along a compact manifold. This ensures both convergence (assuming of course η smooth) and the possibility of exchanging $\pi _{2*}$ and derivatives along Σ. Up to a sign, we then have, by Stokes’ theorem,

$$ {\textrm{d}}_\Sigma \pi _{2*} - \pi _{2*} {\textrm{d}}_{C_2(\Sigma )}=\pi ^\partial _*, $$

where $\pi ^\partial$ is the projection $\partial C_2(\Sigma )\rightarrow \Sigma$. The boundary $\partial C_2(\Sigma )$ can be identified with the unit tangent bundle of Σ.

The analogue of having harmonic forms defined by the metric is now a choice of basis $([\chi _i])_i$ for $H^\bullet (\Sigma )$ with a choice of representatives $\chi _i\in \Omega ^\bullet (\Sigma )$. We denote by

$$ \sigma _{ij} :=\int _\Sigma \chi _i\,\chi _j $$

the entries of the nondegenerate pairing on $H^\bullet (\Sigma )$ in the basis $([\chi _i])_i$. We will denote by $\sigma ^{ij}$ the entries of its inverse.

The soft propagator η is then defined as a smooth 1-form on $C_2(\Sigma )$ satisfying the following properties:

1. ${\textrm{d}}\eta =\pi ^*\chi$, where π is the projection $C_2(\Sigma )\rightarrow \Sigma \times \Sigma$, and χ is the representative of the Euler class of Σ given by
  $$ \chi = -\sum _{ij} \pi _1^*\chi _i\,\pi _2^*\chi _j\,\sigma ^{ij} $$
  with $\pi _1$ and $\pi _2$ the projections $\Sigma \times \Sigma \rightarrow \Sigma$.
2. The restriction of η to $\partial C_2(\Sigma )$ is a global angular form. In particular, $\int _{\partial C_2(\Sigma )}\eta =1$.

In terms of (5.4), condition (1) corresponds to the term $\pi _{{\textrm{Harm}}}$, while condition (2) corresponds to the term $\textrm{id}$. It is shown in [17] (see also [19] for a review) how to construct such an η. Moreover, one can always ensure that $T^*\eta =\eta$, where *T* is the map that exchanges the two points.

##### Remark 5.2

([6, 7]). The Axelrod and Singer’s propagator *K*, see (5.3), also has a kernel η satisfying these properties.

#### Feynman diagrams

The (soft) propagator kernel η can also be identified with the 2-point expectation value (in the theory defined by $\mathcal {J}$) of the fluctuations $\widehat{a}$ and $\widehat{b}$ and therefore with the edges in the Feyman-diagram expansion. More precisely, accordingly to (5.2), we write

$$ \widehat{a} = \underline{a} + \widetilde{a},\qquad \widehat{b} = \underline{b} + \widetilde{b}, $$

with $\underline{a} = \sum _i z^i \chi _i$ and $\underline{b} = \sum _i w^i \chi _i$, where $(z^i,w^i)$ are coordinates on the finite-dimensional BV manifold $H^\bullet (\Sigma )[1]$ with respect to the basis $([\chi _i])_i$. The BV form is just $\omega _{H^\bullet (\Sigma )[1]} = \sum _{ij} {\textrm{d}}z^i\,{\textrm{d}}w^j\,\sigma _{ij}$. Since the kinetic term $\int _\Sigma \widehat{b}\,{\textrm{d}}_{A_0} \widehat{a}$ is just $\int _\Sigma \widetilde{b}\,{\textrm{d}}\widetilde{a}$, we now have

$$ \eta = \langle \pi _1^*\widetilde{a}\,\pi _2^*\widetilde{b}\rangle _0 $$

with $\pi _1$ and $\pi _2$ the projections $C_2(\Sigma )\rightarrow \Sigma$ and $\langle \ \rangle _0$ the expectation value in the unperturbed theory (as such, η corresponds to the other definition of propagator as the inverse of the quadratic form in the action).

The Feynman diagrams of the theory are now obtained, as usual, by recognizing in the expansion of $\mathcal {J}[a,b;\mathcal {A},\mathcal {B}]$ the vertices in $\widetilde{a}$ and $\widetilde{b}$, depending parametrically on $(\underline{a}, \underline{b}, \mathcal {A},\mathcal {B})$. To each vertex we then associate a point on Σ, to be integrated, and to each possible pairing of an $\widetilde{a}$ with a $\widetilde{b}$ we associate a propagator η. Naively each Feynman diagram yields an integral over a product of *n* copies of Σ, where *n* is the number of vertices. The only mild regularization needed here consists in restricting the integration to the open coniguration space $C_n^0(\Sigma )$ obtained by removing from $\Sigma ^n$ all diagonals.

The crucial point is that there exists a nice compactification $C_n(\Sigma )$ of $C_n^0(\Sigma )$, due to Axelrod and Singer [6, 7], elaborating on [43], (see also [18] and [70]), with the following properties:

1. Each $C_n(\Sigma )$ is a smooth compact manifold with corners, with $C_2(\Sigma )$ defined as above.
2. Each projection $C_n^0(\Sigma )\rightarrow C_k^0(\Sigma )$, k<n, corresponding to a choice of *k* points, extends to a smooth map $C_n(\Sigma )\rightarrow C_k(\Sigma )$ of manifolds with corners.

As the boundary of $C_n(\Sigma )$ has measure zero, we can replace the integration on $C_n^0(\Sigma )$ with an integration on $C_n(\Sigma )$. The integrand is now a smooth form obtained as a wedge product of pullbacks $\pi _{ij}^*\eta$ and $(\pi _i^*\underline{a}, \pi _i^*\underline{b}, \pi _i^*\mathcal {A},\pi _i^*\mathcal {B})$. Here $\pi _{ij}:C_n(\Sigma )\rightarrow C_2(\Sigma )$ is the projection corresponding to a pair of vertices *i* and *j* with an $\widetilde{a}$ on the first and a $\widetilde{b}$ on the second joined to produce a propagator; $\pi _i:C_n(\Sigma )\rightarrow C_1(\Sigma )=\Sigma$ is the projection corresponding to a vertex *i* where one of the forms $(\underline{a}, \underline{b}, \mathcal {A},\mathcal {B})$ is placed.

As we are now left with the integral of a smooth form on a compact manifold with boundary, convergence of the integral is ensured.

Moreover, there is a standard technique to obtain the properties of the Feynman expansion, showing that we really get an observable for the ambient *BF* theory coupled to the residual fields $(\underline{a}, \underline{b})$, which uses Stokes theorem. Namely, we have the

##### Proposition 5.3

The perturbative computation of $\mathcal {O}_\sigma (\underline{a}, \underline{b}, \mathcal {A},\mathcal {B})$ is a quantum BV observable for *BF* theory coupled with the surface residual fields, i.e,

$$ Q\mathcal {O}_\sigma -{\textrm{i}}\hbar \Delta \mathcal {O}-{\textrm{i}}\hbar \Delta _\textrm{res}\mathcal {O}=0, $$

where *Q* and Δ refer to *BF* theory (fields $\mathcal {A},\mathcal {B}$), and $\Delta _\textrm{res}$ refers to the residual fields $(\underline{a}, \underline{b})$. In the absence of residual fields, we simply get

$$ Q\mathcal {O}_\sigma -{\textrm{i}}\hbar \Delta \mathcal {O}=0. $$

We postpone the proof to Sect.  6.1.2 (for the case without residual fields) and to Remark 6.4 (for the case with residual fields).

## 2-Knot Invariants

In this section, we describe how to use the surface observables in *BF* theory to obtain invariants of 2-knots.

Let *M* be a closed four-manifold. A 2-knot in *M* is an embedding $\sigma :\Sigma \rightarrow M$, with Σ a closed two-manifold. The expectation value

$$ I_\sigma :=\langle \mathcal {O}_\sigma \rangle $$

is then well-defined and should yield an invariant under isotopies (see Sect.  4.1).

To be precise, we have to remember that the perturbative construction of $\mathcal {O}_\sigma$ yields a half-density on the space of the residual fields for the 2D-*BF*-theory on Σ parametrized by the ambient 4D-*BF*-theory fields and the 2D-*BF*-theory background fields (see Remark 5.1).

The perturbative evaluation of $I_\sigma$ is obtained by integrating out the fluctuations of the ambient-*BF*-theory fields around their own background and residual fields (the derived tangent space at a critical point). At the end, we do not get a numerical invariant: for a fixed choice of background fields,^19^*I* is a map from the space K(Σ,M) of embeddings $\Sigma \rightarrow M$ to the space of solutions of the quantum master equations on the BV space $\mathcal {F}_{\Sigma ,\text {res}}\times \mathcal {F}_{M,\text {res}}$:

$$ I:K(\Sigma ,M) \rightarrow \mathcal {D}^{\frac{1}{2}}(\mathcal {F}_{\Sigma ,\text {res}}\times \mathcal {F}_{M,\text {res}}),\qquad \triangle K_\sigma =0\ \forall \sigma \in K(\Sigma ,M), $$

where $\mathcal {D}^{\frac{1}{2}}$ denotes the space of half-densitites and ▵ is the canonical BV Laplacian.^20^

### Remark 6.1

*(The invariant)* Under an isotopy of σ, $I_\sigma$ changes by a ▵-exact term. At the end of the day, the 2-knot invariant takes values in the quantum BV cohomology of $\mathcal {F}_{\Sigma ,\text {res}}\times \mathcal {F}_{M,\text {res}}$.

### Remark 6.2

*(The trivial case)* In the trivial case studied in the previous section, we have the identification

$$ \mathcal {F}_{\Sigma ,\text {res}}\times \mathcal {F}_{M,\text {res}}\simeq (H^\bullet (\Sigma )[1]\oplus H^\bullet (\Sigma )))\otimes {\mathfrak {g}}\times (H^\bullet (M)[1]\oplus H^\bullet (M)[2]))\otimes {\mathfrak {g}}. $$

Once the spaces of residual fields have been identified and a gauge fixing has been chosen, $I_\sigma$ can be written (more precisely, defined in perturbation theory) as a sum over Feynman diagrams: $I_\sigma = \sum _\Gamma c_\Gamma \int _{\mathcal {C}_\Gamma } \omega _\Gamma$, where $c_\Gamma$ is a numerical coefficient containing the combinatorial contribution, and $\omega _\Gamma$ is a differential form on the configuration space $\mathcal {C}_\Gamma$ of points corresponding to the vertices of the diagram Γ. Note that $\mathcal {C}_\Gamma$ depends on the embedding σ. In the trivial situation, analyzed in the following, the Lie algebra contributions can be stored in $c_\Gamma$, but in general, for a nontrivial principal bundle, they are in $\omega _\Gamma$.

### Long 2-knots

A simpler situation where we can give more explicit formulae and get a numerical invariant is the one considered in [33] (only for the case λ=0). Namely, we take $M={\mathbb {R}}^4$, $\Sigma ={\mathbb {R}}^2$, and we fix a reference embedding $\sigma _0:{\mathbb {R}}^2\rightarrow {\mathbb {R}}^4$, e.g.,

$$ \sigma _0(x,y)=(x,y,0,0). $$

A long 2-knot is an embedding $\sigma :{\mathbb {R}}^2\rightarrow {\mathbb {R}}^4$ that outside a compact subset is equal to $\sigma _0$.

To compute invariants of long 2-knots, we resort to the expectation value of $\mathcal {O}_\sigma$ in *BF* theory. Since now *M* and Σ are not closed manifolds, we have to impose appropriate conditions at infinity for the fields.

#### The construction of $\mathcal {O}_\sigma$

We start with a discussion of the 2D *BF* theory. With the exception of β, we require all the BV fields to vanish at infinity. In order to get an interesting result, we instead require β to converge at infinity to $\hbar \xi$, where ξ is an element of the Lie algebra ${\mathfrak {g}}$ chosen once and for all (it becomes part of the definition of the observable). Accordingly, we choose the background fields as^21^

$$ \alpha _0=0,\qquad \beta _0=\hbar \xi ,\qquad A_0=0,\qquad B_0=0. $$

The decomposition (5.1) then becomes

$$ \begin{aligned} a&= \widehat{a},\\ b&= \hbar \xi + \widehat{b},\\ \mathcal {A}&= \widehat{\mathcal {A}},\\ \mathcal {B}&= \widehat{\mathcal {B}}, \end{aligned} $$

with all the hatted fields vanishing at infinity. In the topology under consideration, there are no residual fields, so, actually,

$$ \widehat{a} = \widetilde{a},\qquad \widehat{b} = \widetilde{b},\qquad \widehat{\mathcal {A}} = \widetilde{\mathcal {A}},\qquad \widehat{\mathcal {B}} = \widetilde{\mathcal {B}}. $$

In summary, we can rewrite the BV surface action as

$$\begin{aligned}&\mathcal {J}_\xi [\widetilde{a},\widetilde{b};\widetilde{\mathcal {A}},\widetilde{\mathcal {B}}]\\&= \int _{{\mathbb {R}}^2}\left( \widetilde{a}{\textrm{d}}\widetilde{b} + \widetilde{a} [\widetilde{\mathcal {A}},\widetilde{a}] + \frac{\lambda }{2} \widetilde{b}[\widetilde{a},\widetilde{a}]+\widetilde{b}\widetilde{\mathcal {B}} +\hbar \xi [\widetilde{\mathcal {A}},\widetilde{a}] + \frac{\lambda }{2}\hbar \xi [\widetilde{a},\widetilde{a}]+\hbar \xi \widetilde{\mathcal {B}} \right) . \end{aligned}$$

From this we can read propagator and vertices for the surface theory.

We start with the propagator. The discussion is as in Sect.  5.1 with some simplifications. The first is that there are no residual fields. The second is that we can give a very explicit formula for the propagator kernel η of Sect.  5.1.2, analogously to [9, 18, 45]. Namely, we consider the map

$$ \phi :\begin{array}{ccc} C_2^0({\mathbb {R}}^2) & \rightarrow &  S^1\\ (x,y) & \mapsto & \frac{x-y}{\Vert x-y\Vert } \end{array} $$

and get $\eta =\frac{1}{2\pi }\phi ^*{\textrm{d}}\vartheta$, with $\vartheta \in [0,2\pi ]$. In Feynman diagrams, this propagator, viewed as the expectation value $\langle \widetilde{a}\widetilde{b}\rangle$, will be denoted by .

As for the vertices, first we have $\hbar \xi B$ with no propagators attached. Then we have the vertices independent of λ:

**Figure:**
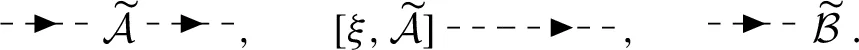

Finally, the vertices with λ are

**Figure:**
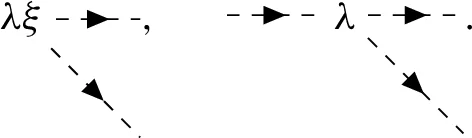

We may now present some Feynman diagrams contributing to $\mathcal {O}_\sigma$.^22^ At λ=0, we only have the diagrams studied in [33, 66]: the “snakes” of various length

**Figure:**
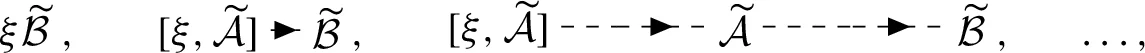

and the “wheels,” also of various length,

**Figure:**
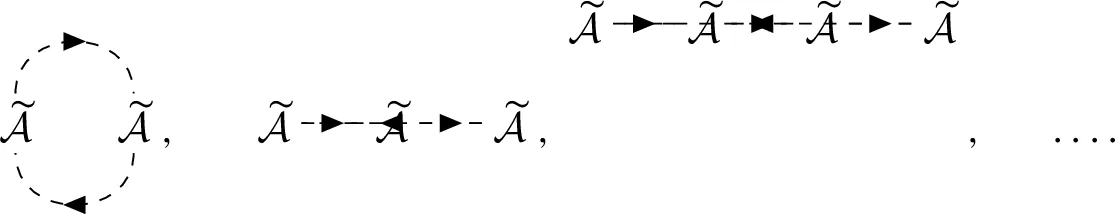

If $\lambda \not =0$, we also have other diagrams. For esample, a snake might start bifurcating

**Figure:**
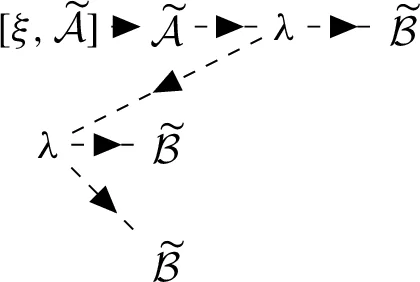

or a wheel might start growing branches

**Figure:**
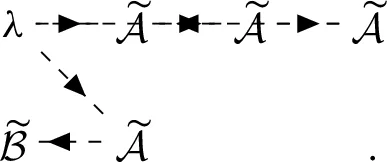

Moreover, we will have diagrams starting at a $\lambda \xi$ vertex, e.g.,

**Figure:**
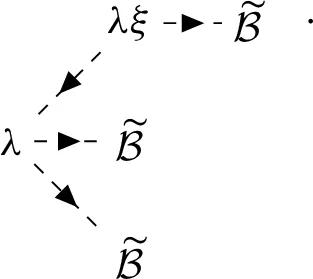

##### Remark 6.3

It is possible to show that the degree in ħ of the Feynman diagrams is equal to the sum of the degree in ξ and the degree in λ:

$$ \deg _\hbar = \deg _\xi + \deg _\lambda . $$

The only diagram of degre 1 in ħ is the vertex $\xi \widetilde{\mathcal {B}}$. Therefore,

6.1 $$\begin{aligned} \mathcal {O}_\sigma = \textrm{e}^{{\textrm{i}}\xi \int _{{\mathbb {R}}^2}\mathcal {B}+\, \cdots }. \end{aligned}$$

Note that, thanks to the invariant pairing on ${\mathfrak {g}}$, we may also think of ξ as an element of ${\mathfrak {g}}^*$ (this is actually the canonical choice in the case of pure *BF* theory, i.e., when λ=0). We may therefore think of ξ as the analogue of choosing a representation of ${\mathfrak {g}}$ in the definition of a Wilson loop.

#### Proof of Proposition 5.3 in the absence of residual fields

We want to prove $Q\mathcal {O}_\sigma -{\textrm{i}}\hbar \Delta \mathcal {O}_\sigma =0$. We now denote the fields by $\widetilde{\mathcal {A}},\widetilde{\mathcal {B}}$, as they are actually the perturbations around the trivial connection and the zero *B* field. In this setting, the cohomological vector field *Q* is better expanded as $Q=Q_0+Q_\textrm{int}$ with

$$ \begin{aligned} Q_0\widetilde{\mathcal {A}}&= {\textrm{d}}\widetilde{\mathcal {A}},\\ Q_0\widetilde{\mathcal {B}}&= {\textrm{d}}\widetilde{\mathcal {B}},\\ Q_\textrm{int}\widetilde{\mathcal {A}}&= \frac{1}{2}[\widetilde{\mathcal {A}},\widetilde{\mathcal {A}}]-\lambda \widetilde{\mathcal {B}},\\ Q_\textrm{int}\widetilde{\mathcal {B}}&= [\widetilde{\mathcal {A}},\widetilde{\mathcal {B}}]. \end{aligned} $$

In the perturbative expansion, $\mathcal {O}_\sigma (\widetilde{\mathcal {A}},\widetilde{\mathcal {B}})$ is the exponential of the sum ${\mathfrak o}_\sigma (\widetilde{\mathcal {A}},\widetilde{\mathcal {B}})$ of the contributions of the just displayed connected Feynman diagrams. To such a diagram Γ, we associate an inhomogeneous differential form $\omega _\Gamma (\mathcal {A},\mathcal {B})$ on the compactified configuration space $C_n(\Sigma )$, with *n* the number of vertices of Γ, according to the rules just displayed. The contribution of Γ is $c_\Gamma \int _{C_n(\Sigma )}\omega _\Gamma (\widetilde{\mathcal {A}},\widetilde{\mathcal {B}})$, where the integral selects the form component of degree equal to the dimension of $C_n(\Sigma )$, and $c_\Gamma$ is a numerical coefficient containing the combinatorial contribution. The core of the proof of Proposition 5.3 consists in the Stokes formula

$$ \int _{C_n(\Sigma )}{\textrm{d}}\omega _\Gamma (\widetilde{\mathcal {A}},\widetilde{\mathcal {B}})= \int _{\partial C_n(\Sigma )}\omega _\Gamma (\widetilde{\mathcal {A}},\widetilde{\mathcal {B}}), $$

where $\partial C_n(\Sigma )$ denotes the codimension-one boundary stratum.

In the absence of residual fields, the propagator η is a closed form (see Section 6.1.1), so ${\textrm{d}}\omega _\Gamma (\widetilde{\mathcal {A}},\widetilde{\mathcal {B}})$ is obtained by ${\textrm{d}}$ acting on $\widetilde{\mathcal {A}}$ and $\widetilde{\mathcal {B}}$. Therefore, we have

$$ \int _{C_n(\Sigma )}{\textrm{d}}\omega _\Gamma (\widetilde{\mathcal {A}},\widetilde{\mathcal {B}})=Q_0\int _{C_n(\Sigma )}\omega _\Gamma (\widetilde{\mathcal {A}},\widetilde{\mathcal {B}}). $$

As usual, we decompose $\partial C_n(\Sigma )$ into the principal faces (corresponding to the collapse of exactly two points) and hidden faces (the remaining ones). The usual vanishing theorems [18, 51] may be applied here, so the only hidden face that might potentially contribute is the one where all points collapse. In this case, the face is a bundle over Σ. The contribution is an integral over Σ with integrand the product of the $\widetilde{\mathcal {A}},\widetilde{\mathcal {B}}$ fields that appeared in the graph (all evaluated at the same point) and the result of the fiber integral. The form to be integrated on the fiber is determined by the graph with all the $\widetilde{\mathcal {A}},\widetilde{\mathcal {B}}$ labels removed. As such, the graph will have at least one vertex that is bivalent or univalent. Again, by the vanishing theorems, the integral vanishes.

Each principal face is a bundle over $C_{n-1}(\Sigma )$, with one of the points corresponding to the collapse and with fiber the sphere tangent bundle at that point. The fiber integral vanishes unless the two collapsing points are connected by a propagator, in which case the integral is one. Therefore, we have

$$ \int _{\partial C_n(\Sigma )}\omega _\Gamma (\widetilde{\mathcal {A}},\widetilde{\mathcal {B}})=\int _{C_{n-1}(\Sigma )}\omega _{\partial \Gamma }(\widetilde{\mathcal {A}},\widetilde{\mathcal {B}}), $$

where $\partial \Gamma$ is the sum of graphs obtained from Γ by collapsing the edges one by one, with appropriate signs coming from the orientation of $\partial C_n(\Sigma )$. The differential form $\omega _{\partial \Gamma }$ is defined accordingly. It is easier and more transparent to describe the contributions to $\omega _{\partial \Gamma }$ starting from $\omega _\Gamma$ and directly studying the principal-face contributions. A graph in the sum $\partial \Gamma$ has dashed edges as before and vertices labeled by λ, ξ, $\widetilde{\mathcal {A}}$, and $\widetilde{\mathcal {B}}$. The difference is that some new vertices now appear. We have: vertices with two $\widetilde{\mathcal {A}}$s (more precisely, $[\widetilde{\mathcal {A}},\widetilde{\mathcal {A}}]$ and $\xi [\widetilde{\mathcal {A}},\widetilde{\mathcal {A}}]$); vertices with one $\widetilde{\mathcal {A}}$ and one $\widetilde{\mathcal {B}}$ (more precisely, $[\widetilde{\mathcal {A}},\widetilde{\mathcal {B}}]$ and $\xi [\widetilde{\mathcal {A}},\widetilde{\mathcal {B}}]$); vertices with one λ and one $\widetilde{\mathcal {B}}$ (more precisely, $\lambda \widetilde{\mathcal {B}}$ and $\lambda [\xi ,\widetilde{\mathcal {B}}]$); vertices with one λ and one $\widetilde{\mathcal {A}}$ (more precisely, $\lambda \widetilde{\mathcal {A}}$); vertices with $\lambda ^2$. When taking the sum over all Γs in ${\mathfrak o}_\sigma (\widetilde{\mathcal {A}},\widetilde{\mathcal {B}})$, the contributions of the last two type of vertices cancel out by the usual Lie algebra arguments (or IHX relations in graphs). The remaining vertices amount to replacing the $\widetilde{\mathcal {A}}$ fields, one by one, by $\frac{1}{2}[\widetilde{A},\widetilde{A}]-\lambda \widetilde{\mathcal {B}}$ and the $\widetilde{\mathcal {B}}$ fields, one by one, by $\frac{1}{2}[\widetilde{A},\widetilde{B}]$. This is the same effect as by acting with $Q_\textrm{int}$.

In summary, we have $Q{\mathfrak o}_\sigma (\widetilde{\mathcal {A}},\widetilde{\mathcal {B}})=0$ and, since *Q* is a derivation, $Q\mathcal {O}_\sigma (\widetilde{\mathcal {A}},\widetilde{\mathcal {B}})=0$.^23^

This shows that $\mathcal {O}_\sigma (\widetilde{\mathcal {A}},\widetilde{\mathcal {B}})=0$ is a classical BV observable. To check that is also a quantum BV observable, we have to apply the BV Laplacian Δ, defined in terms of the fields $\widetilde{\mathcal {A}},\widetilde{\mathcal {B}}$, and show that $\Delta {\mathfrak o}_\sigma +\frac{1}{2}(\mathfrak o_\sigma ,\mathfrak o_\sigma )=0$. This part of the proof is rather formal because we have not regularized Δ yet. What simplifies the argument is however the fact that ${\mathfrak o}_\sigma (\widetilde{\mathcal {A}},\widetilde{\mathcal {B}})$ is multilocal, so the action of Δ may simply be computed by identifying an $\widetilde{\mathcal {A}}$ vertex with a $\widetilde{\mathcal {B}}$ vertex, which does not lead to any singular term.^24^ In particular, applying Δ to a graph containing a $\widetilde{\mathcal {B}}$ produces a new loop, based at a vertex with no *BF* fields. Again, the combinatorics that leads to the vanishing of $\Delta {\mathfrak o}_\sigma +\frac{1}{2}(\mathfrak o_\sigma ,\mathfrak o_\sigma )$ is ensured by the formal BV computation.

##### Remark 6.4

*(The case with residual fields)*. The proof of Proposition 5.3 in the general case proceeds as above, with Stokes theorem as its central element. We quickly list the main differences. First, we have to expand around some reference fields $A_0$ and $B_0$, which we take as critical points of pure *BF* theory (so $A_0$ is flat and $B_0$ is covariantly closed). The BRST operator for *BF* theory will still be decomposed as $Q_0+Q_\textrm{int}$, with $Q_0$ determined by the action of ${\textrm{d}}_{A_0}$. At this level the fluctuations $\widetilde{\mathcal {A}},\widetilde{\mathcal {B}}$ still contain also the *BF* residual fields. Next, in the perturbative evaluation of $\mathcal {O}_\sigma$, we expand around the quadratic term $\beta {\textrm{d}}_{A_0}\alpha$. The residual fields are given by the cohomology of ${\textrm{d}}_{A_0}$ on surface forms. The propagator η is no longer a closed form, yet it satisfies ${\textrm{d}}_{A_0}\eta + \sum _i \pi _1^*\chi _i\pi _2^*\chi ^i$, where the χs are forms representing a basis and the dual basis in the residual fields and $\pi _{1,2}$ are the projections $C_2(\Sigma )\rightarrow \Sigma$ [29, Section 3.3.2]) (see also [25, Section 5.3] and [27, Section 4]). The Feynman diagrams are also decorated by χs: more precisely, a zero mode in *a* gives rise to a term $\sum _i z^i\chi _i$ and a zero mode in *b* to a term $\sum _i z^+_i\chi ^i$; the variables $z,z^+$ are the Darboux coordinates for the space of residual fields. The first part of the argument above now changes because of the extra contribution in ${\textrm{d}}_{A_0}\omega _\Gamma$ due to η’s not being closed. The extra terms are described by cutting an edge and placing χs on the two newly created vertices. The same kind of contribution appears when applying the residual-fields BV Laplacian $\Delta _\textrm{res}=\sum _i\frac{\partial ^2}{\partial z^i\partial z^+_i}$. The rest of the proof is exactly the same: the combinatorics of the principal faces works by the formal BV computation (see also [27, 29]) and the hidden faces do not contribute by the vanishing theorems.

#### The expectation value of $\mathcal {O}_\sigma$

The BV action for the 4D *BF* theory may now be written as

$$ \mathcal {S}= \int _{{\mathbb {R}}^4} \left( \widetilde{B}\,{\textrm{d}}\widetilde{A} + \frac{1}{2} \widetilde{B}[\widetilde{A},\widetilde{A}] -\dfrac{\lambda }{2}\widetilde{B}\widetilde{B} \right) . $$

The propagator can also be constructed analogously to [9, 18, 45] and to the 2-dimensional discussion in Sects.  5.1.1 and 5.1.2. In the present case, we state the result for $M={\mathbb {R}}^4$ and the Lorenz gauge with respect to the euclidean metric. Namely, we consider the map

$$ \Phi :\begin{array}{ccc} C_2^0({\mathbb {R}}^4) & \rightarrow &  S^3\\ (x,y) & \mapsto & \frac{x-y}{\Vert x-y\Vert } \end{array} $$

and get the propagator $\theta =\Phi ^*\omega$, where ω is the normalized, uniform volume form on $S^3$. In Feynman diagrams, this propagator, viewed as the expectation value $\langle \widetilde{\mathcal {B}}\widetilde{\mathcal {A}}\rangle$, will be denoted by  We have two vertices:

**Figure:**
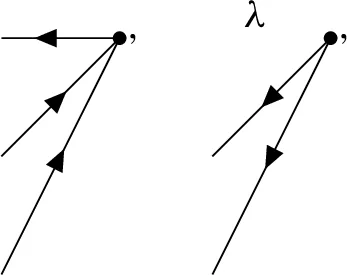

where the bullet are to remember that the vertices are in ${\mathbb {R}}^4$ now.

In the expectation value of $\mathcal {O}_\sigma$, we will then have two type of vertices, corresponding to integration on ${\mathbb {R}}^4$ or on ${\mathbb {R}}^2$, and two type of propagators.^25^

The lowest order in ξ (second order) and no λ is as in [33]. It is the sum of the the three following diagrams.

**Figure:**
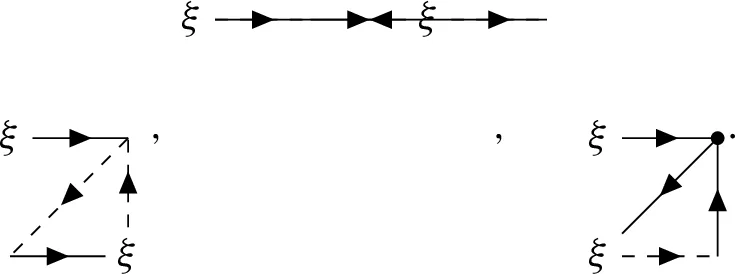

This is also the same as the 2-knot invariant proposed by Bott [16]. It is been proved that the λ-independent diagrams produce invariants related to the coefficients of the Alexander invariant [53, 54, 72]. These invariants should also be related to those constructed in [10].

Switching on λ will produce other invariants, which might be independent from the previous one. We now have several new possibilities. For example, here is a diagram at order $\lambda ^2$ independent of ξ:

**Figure:**
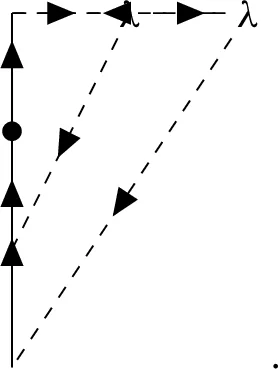

#### Isotopy invariance

According to the heuristics of Sect.  4.1, the expectation value $\langle \mathcal {O}_\sigma \rangle$ should be an isotopy invariant. This result may however be spoiled by some contributions of boundary faces of the compactified configuration spaces (hidden faces), as it also occurs for Chern–Simons knot invariants, for Chern–Simons 3-manifold invariants, and for the long 2-knot invariants of [33, 66]. This violation of an expected property at the quantum level is an example of an anomaly. As in the mentioned cases (see [17, 18, 33, 66]), also here the anomalies can be removed by some universal construction.

Let us first introdue some notation. By $\operatorname {Mon}({\mathbb {R}}^2,{\mathbb {R}}^4)$ we denote the space of linear maps ${\mathbb {R}}^2\rightarrow {\mathbb {R}}^4$. We have the map

$$ T:\begin{array}{ccc} \mathcal {K}\times {\mathbb {R}}^2 & \rightarrow & \operatorname {Mon}({\mathbb {R}}^2,{\mathbb {R}}^4)\\ (\sigma ,{\mathbb {R}}^2) & \mapsto & {\textrm{d}}_x\sigma \end{array} $$

We also denote by $p_1$ the projection map $\mathcal {K}\times {\mathbb {R}}^2$ to the first factor $\mathcal {K}$.

##### Theorem 6.5

The expectation value $\langle \mathcal {O}_\sigma \rangle$ can be corrected to an isotopy invariant

$$ \overline{\Theta }_\sigma :=\langle \mathcal {O}_\sigma \rangle + (p_1)_*T^*\widetilde{\Theta }, $$

where $\widetilde{\Theta }$ is a universal contribution.^26^

Note that $\widetilde{\Theta }$ is defined in terms of corrections $\widetilde{\Theta }_\Gamma$ for the Feynman diagrams Γ appearing in the expansion. For a particular diagram Γ, it may happen that $\widetilde{\Theta }_\Gamma =0$. This is proved by using the vanishing theorems in [66, Section 6.5.1] and symmetries of Γ under permutations of the vertices. For example, for each Γ in the Bott invariant the correction vanishes.

##### Proof

The proof is very similar to that of Proposition 5.3 in Sect.  6.1.2 and replaces the heuristic proof of Sect.  4.1 in the absence of residual fields.

The main difference is that now the Feynman diagrams give rise to integrals on compactified configuration spaces $C_{m,n}^\sigma$ of m+n points in the ambient space with the first *m* points on the image of the embedding σ. These spaces are defined as follows [18]. First observe that an injective map $\sigma :\Sigma \rightarrow M$ defines the map $\sigma _m^0:C_m^0(\Sigma )\rightarrow C_m^0(M)$, $s_1,\dots ,s_m\mapsto \sigma (s_1),\dots ,\sigma (s_m)$. If σ is an embedding, this can be extended to the FMAS compactifications: $\sigma _m:C_m(\Sigma )\rightarrow C_m(M)$. The compactified configuration space $C_{m,n}^\sigma$ is then defined as the fiber product of this map with the projection $C_{m+n}(M)\rightarrow C_m(M)$. As we eventually want to regard the results as functions on the space $\mathcal {K}$ of embeddings, we consider the fiber bundle $\mathcal {C}_{m,n}$ over $\mathcal {K}$ with fiber $C_{m,n}^\sigma$ at σ. Since we are just interested in checking that the final result is locally constant on $\mathcal {K}$, we replace $\mathcal {K}$ by a finite-dimensional manifold, which by abuse of notation we still call $\mathcal {K}$, given by some finite parametrization of $\mathcal {K}$, and check that the differential of the function vanishes for every chosen parametrization.^27^

We now focus on the case $\Sigma ={\mathbb {R}}^2$, $M={\mathbb {R}}^4$, and σ a long 2-knot. A connected Feynman graph Γ defines an inhomogeneous differential form $\omega _\Gamma$ on $C_{m,n}^\sigma$, with m+n the number of vertices of Γ and *n* the number of bulleted vertices, by the prescription above. The contribution of Γ is $c_\Gamma \int _{C_{m,n}^\sigma }\omega _\Gamma$, where the integral selects the form component of degree equal to the dimension of $C_{m,n}^\sigma$, and $c_\Gamma$ is a numerical coefficient containing the combinatorial contribution. Regarded as a function on the space $\mathcal {K}$ of 2-knots, the integral can be written as $\int _{\mathcal {C}_{m,n}}\omega _\Gamma$, as a shorthand notation for the fiber integration in $\mathcal {C}_{m,n}\rightarrow \mathcal {K}$.

The core of the proof now consists in the Stokes formula for fiber integrals

$$ {\textrm{d}}\int _{\mathcal {C}_{m,n}}\omega _\Gamma =\int _{\mathcal {C}_{m,n}}{\textrm{d}}\omega _\Gamma -\int _{\partial \mathcal {C}_{m,n}}\omega _\Gamma , $$

where $\partial \mathcal {C}_{m,n}$ denotes the codimension-one boundary stratum. Since the propagators are closed, this simplifies to

$$ {\textrm{d}}\int _{\mathcal {C}_{m,n}}\omega _\Gamma =-\int _{\partial \mathcal {C}_{m,n}}\omega _\Gamma . $$

We decompose $\partial \mathcal {C}_{m,n}$ into the principal faces (corresponding to the collapse of exactly two points) and hidden faces (the remaining ones). The vanishing theorems of [66, Section 6.5.1] may be applied here, so the only hidden face that might potentially contribute is the one where all points collapse. Its effect was carefully analyzed in [66, Section 6] and gives rise to the anomaly correction $(p_1)_*T^*\widetilde{\Theta }$.^28^

Finally, we consider the principal faces. Each of them is a bundle over $$\partial \mathcal {C}_{m',n'}$$ with $m^{\prime }=m-1,n^{\prime }=n$ or $m^{\prime }=m,n^{\prime }=n-1$. The fiber integral vanishes unless the two collapsing points are connected by a propagator (of either type), in which case the integral is one. Therefore, we have

$$ \int _{\partial \mathcal {C}_{m,n}}\omega _\Gamma =\int _{\partial \mathcal {C}_{m',n'}}\omega _{\partial \Gamma }, $$

where $\partial \Gamma$ is the sum of graphs obtained from Γ by collapsing the edges (of either type) one by one, with appropriate signs coming from the orientation of $\partial \mathcal {C}_{m',n'}$. The differential form $\omega _{\partial \Gamma }$ is defined accordingly. It is easier and more transparent to describe the contributions to $\omega _{\partial \Gamma }$ starting from $\omega _\Gamma$ and directly studying the principal-face contributions. A graph in the sum $\partial \Gamma$ has edges (of the two types) as before and vertices possibly labeled by λ and/or ξ. The difference now is that there will be one special vertex (the result of the contraction) that is different from those we had before. It could be, e.g., a trivalent bullet vertex or a solid bivalent nonbullet vertex or any other vertex resulting from the contraction. When taking the sum over all Γs in $\langle \mathcal {O}_\sigma \rangle$, the contributions cancel out by the usual Lie algebra arguments (or IHX relations in graphs), exactly because signs and combinatorics for principal faces are taken into account by the formal BV computation.

In conclusion, ${\textrm{d}}\overline{\Theta }_\sigma =0$, so $\overline{\Theta }_\sigma$ is locally constant on $\mathcal {K}$ and is therefore an isotopy invariant. □

##### Remark 6.6

*(The general case)* The arguments are similar to those above and in Remark 6.4 and replace the heuristic proof of Sect.  4.1. The main change is that $\langle \mathcal {O}_\sigma \rangle$ is no longer a number but a function on the BV spaces of residual fields (both for the ambient and for the surface theory). The propagators are no longer closed forms. Their exterior derivatives are linear combinations of products of basis elements and the corresponding dual basis elements (realized as differential forms) placed on the two endpoints. The Feynman diagrams contain new vertices labeled by such basis elements times the corresponding coordinates in the BV spaces of residual fields. In the Stokes-theorem computation, at the core of the proof, the extra terms obtained by differentiating are compensated by the terms obtained by applying the BV Laplacian on residual fields (by the mechanism described in [27, Section 4]).

## BV Surface Observables for Yang–Mills Theory

As discovered in [13], 4D (euclidean) Yang–Mills theory can be recovered from *BF* theory with cosmological term via a BV pushforward. This will allow us turning our observable $\mathcal {O}_\sigma$ into a surface observable for Yang–Mills theory.

### Yang–Mills theory from *BF* theory

Let us recall the construction of [13]. We start with the BV action (4.2) for *BF* theory. Next we pick the riemannian metric we want to use to define euclidean Yang–Mills theory and split the field *B* and its antifield $B^+$ into their self-dual and anti-self-dual components:^29^

$$ B=B_++B_-,\qquad B^+=B^+_++B^+_-. $$

Then we split the space $\mathcal {F}$ of BV fields for *BF* theory into the product $\mathcal {F}_1\times \mathcal {F}_2$, where, in the notation of (4.1), we use $(c,A,B^+_+,B_+,A^+,c^+)$ as coordinates for $\mathcal {F}_1$ and $(B^+_-,\tau ^+,\phi ^+,\phi ,\tau ,B_-)$ as coordinates for $\mathcal {F}_2$. If we split the original BV action (4.2),

$$\begin{aligned}&\mathcal {S}= \int _M\left( BF_A -\dfrac{\lambda }{2}BB + A^+({\textrm{d}}_Ac-\lambda \tau ) + B^+([c,B]+{\textrm{d}}_A\tau )\right. \\&\quad \quad \left. +c^+\left( \frac{1}{2}[c,c]-\lambda \phi \right) +\tau ^+([c,\tau ]+{\textrm{d}}_A\phi )+\phi ^+[c,\phi ]+\dfrac{1}{2}[B^+,B^+] \right) , \end{aligned}$$

as $$\mathcal {S}_\text {YM}^\text {1st}+\mathcal {S}_\text {fiber}$$ with

$$ \mathcal {S}_\text {YM}^\text {1st} = \int _M\left( B_+F_A -\dfrac{\lambda }{2}B_+B_+ + A^+{\textrm{d}}_Ac + B^+_+[c,B_+] +c^+\frac{1}{2}[c,c] \right) , $$

we recognize $(\mathcal {F}_1,\omega _1,\mathcal {S}_\text {YM}^\text {1st})$ as the BV triple for Yang–Mills theory in the first-order formalism [22, 23, 36].

#### Proposition 7.1

Ignoring residual fields, there is a choice of gauge-fixing Lagrangian for which the BV pushforward of $\mathcal {S}$ to $\mathcal {F}_1$ yields the effective action $\mathcal {S}_\text {eff}=\mathcal {S}_\text {YM}^\text {1st}+\hbar \, \mathcal {S}_\text {corr}$.

By “ignoring residual fields,” we mean that we set them to zero if they are present (the residual fields contributions can be computed as in [13]). In the following, we will actually focus on the case $M={\mathbb {R}}^4$, and on long 2-knots as in Sect.  6.1, where the residual fields are actually absent.

#### Remark 7.2

For this computation we work around a flat reference connection $A_0$. The residual fields then belong to the cohomology of the covariant exterior derivative ${\textrm{d}}_{A_0}$. A case of particular interest where there are no residual fields is that of $M={\mathbb {R}}^4$ and $A_0=0$—the trivial connection. If residual fields are present, they will give contribution of order 1 to the effective action. One can set them to zero or force them to zero by inserting appropriate observables. We will not discuss this in this paper.

#### Remark 7.3

The quantum corrections in $\mathcal {S}_\text {corr}$ are nonlocal and depend on the chosen riemannian metric. We may view $\mathcal {S}_\text {corr}$ as a cloud correcting Yang–Mills theory at the quantum level. In this version, the surface observable will be obtained directly from *BF* theory via BV pushforward. Alternatively, we may prefer to work with standard Yang–Mills theory and use the cloud $\mathcal {S}_\text {corr}$ to correct the surface observable. See Sect.  7.2 for the full discussion. Clouds appearing in field theory and affecting observables have appeared in a different context, also related to BV pushforward, in [4].

#### Proof of Proposition 7.1

What we have to show is that the Feynman diagrams in the BV pushforward contain no trees and only one-loop diagrams if the residual fields are set to zero.

For the BV pushforward from $\mathcal {F}$ to $\mathcal {F}_1$, we first need the explicit form of $\mathcal {S}_\text {fiber}$:

$$\begin{aligned}&\mathcal {S}_\text {fiber} = \int _M\left( B_-F_A -\dfrac{\lambda }{2}B_-B_- + \lambda A^+\tau + B^+_+{\textrm{d}}_A\tau + B^+_-([c,B_-]+{\textrm{d}}_A\tau )\right. \\&\quad \left. + \lambda c^+\phi +\tau ^+([c,\tau ]+{\textrm{d}}_A\phi )+\phi ^+[c,\phi ]+\dfrac{1}{2}\phi [B^+_-,B^+_-] + \dfrac{1}{2}\phi [B^+_+,B^+_+] \right) . \end{aligned}$$

To perform the BV pushforward over the variables $(B^+_-,\tau ^+,\phi ^+,\phi ,\tau ,B_-)$,^30^ we take the gauge fixing

$$ \phi ^+=0,\qquad B_-=0,\qquad \tau ^+=\tau ^+_\text {gf}\in {{\,\textrm{im}\,}}{\textrm{d}}_{A_0}^*,\qquad \tau =\tau _\text {gf}\in {{\,\textrm{im}\,}}{\textrm{d}}_{A_0}^*, $$

where ${\textrm{d}}_{A_0}^*$ is the formal adjoint of the covariant exterior derivative ${\textrm{d}}_{A_0}$ relative to the background connection $A_0$ we are working around.^31^ The gauge-fixed action becomes

7.1 $$\begin{aligned}&\mathcal {S}_\text {fiber}^\text {gf} = \int _M\left( \phantom {\frac{1}{2}} \lambda A^+\tau _\text {gf} + B^+_+{\textrm{d}}_A\tau _\text {gf} + B^+_-{\textrm{d}}_A\tau _\text {gf} +\lambda c^+\phi \right. \nonumber \\&\quad \left. +\tau _\text {gf}^+([c,\tau _\text {gf}]+{\textrm{d}}_A\phi ) +\dfrac{1}{2}\phi [B^+_-,B^+_-] + \dfrac{1}{2}\phi [B^+_+,B^+_+] \right) . \end{aligned}$$

A very simple observation now is that rescaling $\phi \mapsto \hbar \phi$ and $\tau \mapsto \hbar \tau$ rescales $\mathcal {S}_\text {fiber}^\text {gf}$ by ħ. As a result, $\textrm{e}^{\frac{{\textrm{i}}}{\hbar }\mathcal {S}_\text {fiber}^\text {gf}}$ becomes $\textrm{e}^{{\textrm{i}}\mathcal {S}_\text {fiber}^\text {gf}}$, which is completely independent of ħ. It follows that its BV pushforward, which we denote by $\textrm{e}^{{\textrm{i}}\mathcal {S}_\text {corr}}$ is also independent of ħ. □

#### Remark 7.4

We can interpret the correction term $\textrm{e}^{{\textrm{i}}\mathcal {S}_\text {corr}}$ as a modification to the naive functional measure of Yang–Mills theory.

#### Remark 7.5

*(Gauge transformations)* Since $\mathcal {S}_\text {fiber}^\text {gf}$ depends on $A^+$, the gauge transformations for *A* in $\mathcal {S}_\text {eff}$ are deformed by a term of order ħ.

#### More details on the BV pushforward

It may also be useful to see more in details what kind of Feynman diagrams are generated by the BV pushforward of $\mathcal {S}_\text {fiber}^\text {gf}$. We write $\mathcal {S}_\text {fiber}^\text {gf}=\mathcal {S}_\text {kin}+\mathcal {S}_\text {int}+\mathcal {S}_\text {sou}$ with

$$ \begin{aligned} \mathcal {S}_\text {kin}&= \int _M\left( B^+_-{\textrm{d}}_{A_0}\tau _\text {gf} + \tau _\text {gf}^+{\textrm{d}}_{A_0}\phi \right) ,\\ \mathcal {S}_\text {int}&= \frac{1}{2}\int _M \phi [B^+_-,B^+_-],\\ \mathcal {S}_\text {sou}&= \int _M\left( \lambda A^+\tau _\text {gf} + B^+_+{\textrm{d}}_{A_0}\tau _\text {gf} + B^+_+[\widehat{A},\tau _\text {gf}] + B^+_-[\widehat{A},\tau _\text {gf}] +\lambda c^+\phi \right. \\&\phantom {= \int _M} \left. +\tau _\text {gf}^+[c,\tau _\text {gf}] +\tau _\text {gf}^+[\widehat{A},\phi ] + \dfrac{1}{2}\phi [B^+_+,B^+_+] \right) \end{aligned} $$

with $$\widehat{A} = A-A_0$$.

The terms in $\mathcal {S}_\text {kin}$ produce a determinant (of order 1 and hence a correction of oder ħ to the effective action) and the propagators $\langle B^+_-\ \tau _\text {gf} \rangle$ and $\langle \tau _\text {gf}^+\ \phi \rangle$, both of order ħ. The vertices $B^+_-[\widehat{A},\tau _\text {gf}]$ and $\tau _\text {gf}^+[\widehat{A},\phi ]$ may be used to produce long strings, which we may call fat propagators $\ll B^+_-\ \tau _\text {gf} \gg$ and $\ll \tau _\text {gf}^+\ \phi \gg$, both again of order ħ.

Next we look at the resulting diagrams, which will produce corrections to $\mathcal {S}_\text {YM}^\text {1st}$ in the effective action. One class of diagrams consists in closing a fat propagator, joining the initial with the final vertex. This produces a wheel. As each wheel has order 1, the correction to the effective action will be of order ħ.

The remarkable point is that there are no diagrams at order 1/ħ (so no correction to the effective action at order 1). In fact, we are left with terms linear in $\tau _\text {gf}$, terms linear in ϕ, and the term $\tau _\text {gf}^+[c,\tau _\text {gf}]$ which is bilinear in $\tau _\text {gf}$ and $\tau _\text {gf}^+$. Moreover, the only interaction term is in $\mathcal {S}_\text {int}$. There is no way to construct a tree connecting these vertices by fat propagators.

Thus, all the possible diagrams have at least one loop, which means they are at least at order 1, providing corrections to the effective action of order at least ħ. However, from the combinatorics of the vertices, it is clear we cannot produce more than one loop.

#### From first to second order

The second-order formulation of Yang–Mills theory is easily recovered from $\mathcal {S}_\text {YM}^\text {1st}$ by a further BV pushforward over $(B_+,B^+_+)$. As a gauge-fixing we choose $B^+_+=0$. The gaussian integration over $B_+$ then yields

$$ \mathcal {S}_\text {YM}=-\int _M\left( \frac{1}{2\lambda }(F_A)_+(F_A)_+ - A^+{\textrm{d}}_Ac -c^+\frac{1}{2}[c,c]. \right) $$

This is the BV action for Yang–Mills theory with a specific choice of topological term.^32^ Starting from $\mathcal {S}^\kappa$ as defined in Sect.  4.2.1 yields instead

$$ \int _M\left( \frac{1}{4\lambda }F_A*F_A+\left( \frac{1}{4\lambda }+\frac{\kappa }{2}\right) F_AF_A- A^+{\textrm{d}}_Ac -c^+\frac{1}{2}[c,c] \right) , $$

and we can get whatever theta angle we prefer.

Since we start from the modified action $\mathcal {S}_\text {eff}=\mathcal {S}_\text {YM}^\text {1st}+\hbar \, \mathcal {S}_\text {corr}$, the BV pushforward considered here will produce corrections to $\mathcal {S}_\text {YM}$ at the first order in ħ.

#### Wilson loops

Wilson loops are observables for Yang–Mills theory, both in the first- and in the second-order formalism. They are not observables for the modified theory because the gauge transformations for *A* are changed, see Remark 7.5.

We can remedy for this by dressing the Wilson loop $W_\gamma$ associated to a loop γ (and some representation) to get the BV observable

$$ \mathcal {W}_\gamma :=\textrm{e}^{-{\textrm{i}}\mathcal {S}_\text {corr}} W_\gamma $$

for the BV theory defined by $\mathcal {S}_\text {eff}$.

Note that while $W_\gamma$ only depends on *A*, the BV observable $\mathcal {W}_\gamma$ depends on all the BV fields $(c,A,B^+_+,B_+,A^+,c^+)$. Moreover, $\mathcal {W}_\gamma$ is no longer localized on γ because the correction term $\mathcal {S}_\text {corr}$ is highly nonlocal.

### Surface observables

To get surface observables for Yang–Mills theory, we start from the *BF* theory action $\mathcal {S}$ and from the surface action $\mathcal {J}$. As we know from Sect.  4, we can consider the sum $\widehat{\mathcal {S}}:=\mathcal {S}+\mathcal {J}$ as a BV action. If we then perform the BV pushforward jointly over $\mathcal {F}_2$, as in the last subsection, and over the surface fields *a* and *b*, we get an observable $\mathcal {O}_\sigma ^\text {YM,1st}$ for Yang–Mills theory plus the ħ-correction. We might also decide to integrate only over $\mathcal {F}_2$. In this case, the resulting Yang–Mills observable will still be expressed in terms of a functional integral over surface fields.

To obtain an observable for the standard first-order Yang–Mills theory $\mathcal {S}_\text {YM}^\text {1st}$, we may dress $\mathcal {O}_\sigma ^\text {YM,1st}$ with the correction term. Namely, we define

$$ \widehat{\mathcal {O}}_\sigma ^\text {YM,1st}:=\textrm{e}^{{\textrm{i}}\mathcal {S}_\text {corr}} \mathcal {O}_\sigma ^\text {YM,1st}, $$

which is now a quantum BV observable for $\mathcal {S}_\text {YM}^\text {1st}$. Note that the price we pay is that $\widehat{\mathcal {O}}_\sigma ^\text {YM,1st}$ is no longer localized on the image of σ.

The expectation value of $\mathcal {O}_\sigma ^\text {YM,1st}$ in Yang–Mills theory plus the ħ-correction (or of $\widehat{\mathcal {O}}_\sigma ^\text {YM,1st}$ in the standard first-order Yang–Mills theory) is, by construction, the same as the expectation value of $\mathcal {O}_\sigma$ in *BF* theory, so an invariant of the embedding σ.

#### Remark 7.6

*(Expectation values)*. To get nontopological results, we should compute the expectation value of $\mathcal {O}_\sigma ^\text {YM,1st}$ (possibly for several disjoint embeddings) multiplied by other Yang–Mills observables. Recall however that quantum BV observables do not form an algebra, since the BV Laplacian is a second-order operator. As a consequence the product of the surface observable and, e.g., a Wilson loop is not an observable.^33^ Therefore, electric and magnetic fluxes cannot be measured simultaneously (although by a different mechanism than in the abelian case considered in [41, 67]).

Our last remark is that, under the assumptions of Sect.  6.1, see Eq. (6.1), the Yang–Mills observable will have the form

$$ \mathcal {O}_\sigma ^\text {YM,1st}= \textrm{e}^{{\textrm{i}}\int _\Sigma \xi B_+ + \, \cdots }. $$

If we then go to the second-order formulation, as in Sect.  7.1.2, we get

7.2 $$\begin{aligned} \mathcal {O}_\sigma ^\text {YM,2nd}= \textrm{e}^{\frac{{\textrm{i}}}{\lambda }\int _\Sigma \xi (F_A)_+ + \, \cdots }= \textrm{e}^{\frac{{\textrm{i}}}{2\lambda }\int _\Sigma \xi (*F_A + F_A) + \, \cdots }. \end{aligned}$$

This is a mixture of electric and magnetic observables. We propose two ways to recover a purely electric observable.

#### Electric observable I: Change the initial observable

Instead of $\mathcal {J}$, we may consider the observable $\mathcal {J}_\gamma$ defined in (4.4). In this case, the computations above yield

$$ \mathcal {O}_\sigma ^\text {YM,2nd}= \textrm{e}^{{\textrm{i}}(1-\lambda \gamma )\int _\Sigma \xi \left( \frac{(F_A)_+}{\lambda }- \gamma F_A \right) + \, \cdots }= \textrm{e}^{{\textrm{i}}(1-\lambda \gamma )\int _\Sigma \xi \left( \frac{*F_A}{2\lambda } +\left( \frac{1}{2\lambda }-\gamma \right) F_A\right) + \, \cdots }, $$

so it is enough to choose $\gamma =\frac{1}{2\lambda }$ to get the purely electric observable

$$ \mathcal {O}_\sigma ^\text {YM,2nd,electric}= \textrm{e}^{\frac{{\textrm{i}}}{4\lambda }\int _\Sigma \xi *F_A + \, \cdots }. $$

Note that to achieve this we have to start with

$$ \mathcal {J}_{\frac{1}{2\lambda }} = \frac{1}{2}\int _\Sigma \left( b\,{\textrm{d}}_\mathcal {A}a+ \lambda b[a,a]+b \mathcal {B}-\frac{1}{2\lambda } b F_\mathcal {A}\right) . $$

By the rescaling $b\mapsto \lambda b$, $a\mapsto a/\lambda$, we can convert this to

$$ \mathcal {J}_{\frac{1}{2\lambda }} = \frac{1}{2}\int _\Sigma \left( b\,{\textrm{d}}_\mathcal {A}a+ b[a,a]+\lambda b \mathcal {B}-\frac{1}{2} b F_\mathcal {A}\right) . $$

#### Electric observable II: Divide by a Wilson surface

In [2] a functional-integral Wilson-surface observable for Yang–Mills theory was introduced (motivated by the path-integral presentation of Wilson loops of [3] via the nonabelian Stokes theorem of [38]). At the classical level, the authors also take an embedding σ with image Σ and consider the action

7.3 $$\begin{aligned} Y_\sigma [\alpha ,\beta ;A] :=\int _\Sigma \beta F_{A+\alpha } =\int _\Sigma \left( \beta \,{\textrm{d}}_A\alpha +\frac{1}{2}\beta [\alpha ,\alpha ]+\beta F_A\right) . \end{aligned}$$

We now proceed as in (2.2) and observe that the infinitesimal gauge transformation $\delta A = -{\textrm{d}}_A\gamma$ can be compensated by the infinitesimal change of variables

$$ \alpha \leadsto \alpha -[\gamma ,\alpha ],\quad \beta \leadsto \beta -[\gamma ,\beta ]. $$

Extending this to the BV formalism and computing the BV pushforward over the surface fields yields an observable for Yang–Mills theory.

The problem now is that this is not an observable for the Yang–Mills theory with ħ-correction $\mathcal {S}_\text {eff}$ we obtained by BV pushforward, see Remark 7.5.

However, we may consider $Y_\sigma$ in (7.3) directly in *BF* theory. It is easy to see that the infinitesimal symmetry transformations (2.3) are compensated by the infinitesimal change of variables

$$ \alpha \leadsto \alpha +\lambda \theta -[\gamma ,\alpha ],\quad \beta \leadsto \beta -[\gamma ,\beta ]. $$

Extending this to the BV formalism leads to the BV action

$$ \mathcal {Y}_\sigma [a,b;\mathcal {A},\mathcal {B}] :=\int _\Sigma b F_{\mathcal {A}+ a}= \int _\Sigma \left( b\,{\textrm{d}}_\mathcal {A}a+\frac{1}{2} b[a,a]+b F_\mathcal {A}\right) . $$

Computing the BV pushforward over the surface fields yields an observable $\mathcal {U}_\sigma$ for *BF* theory. Performing the BV pushforward as in Sect.  7.1 finally produces observables $\mathcal {U}_\sigma ^\text {YM,1st}$ and $\mathcal {U}_\sigma ^\text {YM,2nd}$ for Yang–Mills theory in the first- and second-order formalism plus the ħ corrections. Denoting by $\hbar \eta$ the background solution for *b*, and proceeding as in Sect.  7.2, we get

$$ \mathcal {U}_\sigma ^\text {YM,2nd} = \textrm{e}^{{\textrm{i}}\int _\Sigma \eta F_A + \, \cdots }. $$

The rough idea is now to multiply the two $\mathcal {S}_\text {eff}$-observables $\mathcal {O}_\sigma$ and $\mathcal {U}_\sigma$ to get rid of the magnetic part. Comparing with (7.2), we see that we should choose $\eta =-\frac{1}{2\lambda }\xi$ to achieve this. This idea does not work properly because the product of two quantum BV observables is not necessarily a quantum BV observable, as the BV Laplacian is a second-order differential operator.

This problem is however readily removed by a framing of Σ, namely, by displacing the surface Σ in $\mathcal {Y}$ by some nowhere vanishing vector field ϵZ transversal to Σ. The product of $\textrm{e}^{\frac{{\textrm{i}}}{\hbar }\mathcal {J}_\sigma }$ and $\textrm{e}^{\frac{{\textrm{i}}}{\hbar }\mathcal {Y}_{\sigma '}}$ is now a quantum BV observable, and its BV pushforward (with background fields ξ and $\eta =-\frac{1}{2\lambda }\xi$) will produce, in the limit $\epsilon \rightarrow 0$ (to be taken after taking the expectation value), the desired purely electric observable for Yang–Mills theory.

## Self-Dual Yang–Mills Theory

A simpler version of Yang–Mills theory, which fits very well in our description, is self-dual Yang–Mills theory [34, 39, 56, 65].

The BV space of fields $\mathcal {F}_1$ is the same as that for first-order Yang–Mills theory and has coordinates $(c,A,B^+_+,B_+,A^+,c^+)$. The BV action is instead

$$ \mathcal {S}_\text {YM}^\text {sd} = \int _M\left( B_+F_A + A^+{\textrm{d}}_Ac + B^+_+[c,B_+] +c^+\frac{1}{2}[c,c] \right) , $$

which is obtained from first-order Yang–Mills theory by setting λ=0.

All the considerations of the previous sections now apply (with some simplifcation) to the present case. We just have to start from pure *BF* theory and its corresponding surface observable, determined by $\mathcal {J}_\sigma$ with λ=0. We have the analogue of Proposition 7.1.

### Proposition 8.1

Ignoring residual fields, there is a choice of gauge-fixing Lagrangian for which the BV pushforward of $\mathcal {S}$, with λ=0, to $\mathcal {F}_1$ yields the effective action $\mathcal {S}_\text {eff}=\mathcal {S}_\text {YM}^\text {sd}+\hbar \, \mathcal {S}_\text {corr}$, and the surface observable $\mathcal {O}_\sigma$ for pure *BF* theory determines an observable $\mathcal {S}_\text {YM}^\text {sd}$. In turn, the dressing

$$ \widehat{\mathcal {O}}_\sigma ^\text {YM,sd}:=\textrm{e}^{{\textrm{i}}\mathcal {S}_\text {corr}} \mathcal {O}_\sigma ^\text {YM,sd} $$

is an observable for self-dual Yang–Mills theory, with BV action $\mathcal {S}_\text {YM}^\text {sd}$.

The difference is that now $A^+$ does not appear in $\mathcal {S}_\text {fiber}$, see (7.1), so neither in $\mathcal {S}_\text {corr}$, which means that the gauge transformations for *A* in $\mathcal {S}_\text {eff}$ are the usual ones, determined by $\mathcal {S}_\text {YM}^\text {sd}$. As a consequence, not only $\mathcal {O}_\sigma ^\text {YM,sd}$ is an observable but also a Wilson loop $W_\gamma$ is so.

Since $W_\gamma$ only depends on *A*, which is a field in $\mathcal {F}_1$, and since it is an observable also for pure *BF* theory, we can view it as a BV pushforward of itself.

If γ and the image of σ do not intersect, the product $\mathcal {O}_\sigma W_\gamma$ is a quantum BV observable for pure *BF* theory, and its BV pushforward is the quantum BV obervable $\mathcal {O}_\sigma ^\text {YM,sd} W_\gamma$ in the modified self-dual Yang–Mills theory determined by $\mathcal {S}_\text {eff}$.

Finally, $\widehat{\mathcal {O}}_\sigma ^\text {YM,sd} W_\gamma$ is a quantum BV observable for self-dual Yang–Mills theory, and its expectation value is a topological invariant.

## Data Availability

The manuscript has no associated data.

## Footnotes

1 In the setting we will consider explicitly (long 2-knots), ξ is a condition at infinity for one the fields living on the surface and, as such, it is a chosen constant. In the case of a closed surface, which we consider but will not work out explicitly, ξ is instead a classical solution and, as such, it is a covariantly constant function on the surface. In principle, one should then integrate over the space of such solutions or fix one of them by “soaking zero modes.”

2 More precisely, ’t Hooft operators are introduced as duals to Wilson loops. In the surface version, they meausure the electric flux, like the surface version of Wilson loops measures the magnetic flux. Properly speaking, an ’t Hooft operator is related to our construction but with a surface with boundary, which we do not consider in this note but deserves further study.

3 If the manifold is not compact, suitable vanishing conditions of the fields are assumed.

4 The invariant pairing is actually not needed in this setting, for we can define *B* to be a 2-form in the coadjoint bundle $\textrm{ad}^*P$ (or, in the simplified case when *P* is trivial, as an element of $\Omega ^2(M)\otimes {\mathfrak {g}}^*$). In this case, $BF_A$ is a shorthand notation for $(B,F_A)$, where $(\, , \, )$ is the canonical pairing. We prefer to use the version with the invariant pairing because it fits the extension we are going to consider next.

5 If ${\mathfrak {g}}$ is semisimple compact, we may take $\langle \, , \, \rangle$ to be the Killing form. In this case, the convention is just that the trace symbol is understood (or defined to be part of the integral sign $\int _M$).

6 Our convention is to define the hamiltonian vector field $X_g$ of a function *g* by $\iota _{X_g}\omega =\delta g$ and the BV bracket by $(g,h)=(-1)^{g+1}X_gh$.

7 A half-density is locally a function but transforms from chart to chart with the square root of the Berezinian of the transformation. As a consequence, its square can be integrated, up to convergence issues.

8 The existence of such a ρ in the finite-dimensional case is a consequence of the fact that an odd symplectic manifold is always symplectomorphic, although in a noncanonical way, to an odd cotangent bundle, $\mathcal {F}\cong T^*[-1]M$ with *M* an ordinary oriented manifold [68]. Any volume form on *M* provides such a reference half-density.

9 In physics, one often realizes $\mathcal {L}_t$ as the graph in $T^*[-1]\mathcal {M}$ of an odd function $\Psi _t$ of ghost number -1—the gauge-fixing fermion.

10 In [29] the case of nontrivial fibrations is also discussed.

11 To be precise, it is well-defined assuming that the integral converges.

12 In particular, we get

$$ \begin{aligned} QA&= {\textrm{d}}_Ac-\lambda \tau ,\\ QB&= {\textrm{d}}_A\tau +[c,B]+[B^+,\phi ]. \end{aligned} $$

As a result of our sign conventions, we then have, e.g., $Q F_A=-{\textrm{d}}_A\delta A = -[F_A,c] + \lambda \, {\textrm{d}}_A\tau$. Consistency with (2.3) is recovered by setting $B^+$ to zero and using the relations

$$ Q = \epsilon \delta _{\gamma ,\theta },\quad c=\epsilon \gamma , \quad \tau = \epsilon \theta , $$

with ϵ an odd variable.

13 In particular, if *P* is trivial, we may choose $A_0$ as the zero connection and get $\mathcal {F}_M=(\Omega ^\bullet (M)[1]\oplus \Omega ^\bullet (M)[2])\otimes {\mathfrak {g}}$.

14 In principle, a critical point for the theory is a quadruple $(A_0,B_0,\alpha _0,\beta _0)$ satisfied the coupled equations; yet we want to think of $\mathcal {J}$ as defining an observable for the ambient theory, so we first look for a critical point $(A_0,B_0)$ for the ambient theory that ignores $\mathcal {J}$ and then a related critical point $(\alpha _0,\beta _0)$. The choice of $(A_0,B_0,\alpha _0,\beta _0)$ is only used to start the pertubative expansion, and we will see below that this is indeed well-defined for our choice.

15 This corresponds to the Lorenz gauge in physics. We choose a riemannian metric (instead of a lorentzian one) because we need the Hodge decomposition to get a strong deformation retract with finite-dimensional space of residual fields and a nice regularization of Feynman integrals in terms of compactified configuration spaces.

16 Axelrod and Singer’s propagator has further nice properties, sometimes called the side conditions of an SDR (strong/special deformation retract):

$$ K^2=0,\qquad K\iota =0,\qquad \pi _{{\textrm{Harm}}}K=0. $$

We will not demand them in general, even though they may be useful for more explicit computations. Also note that a soft propagator in general does not correspond to a propagator for a BV gauge fixing, since this would satisfy the side conditions. On the other hand, soft propagators are easier to construct and to handle. The general techniques on compactified configurations spaces (see Sects.  6.1.2 and Remark 6.1.4), show that the expectation values of observables are indepedent of the choice of soft propagator.

17 In general, for Σ of dimension *k*, η is a (k-1)-form.

18 In practice, $C_2(\Sigma )$ is easily described in terms of coordinates: the usual ones from $\Sigma \times \Sigma$ away from the diagonal, whereas near the diagonal we take polar coordinates (r,θ) for one of the two points in Σ with respect to the other and compactify by allowing *r* also to attain the value zero.

19 One could further work in the direction of globalization relating *I* to a globally well-defined density on the space of background fields, along the lines of [14, 24, 32]. We will not pursue this here.

20 Recall (Remark 5.1) that $\mathcal {O}_\sigma$ is already a half-density on $\mathcal {F}_{\Sigma ,\text {res}}$. The perturbative computation of the gauge-fixed BV integral $\langle \mathcal {O}_\sigma \rangle$ over the fluctuations of the ambient *BF* theory yields a half-density on $\mathcal {F}_{M,\text {res}}$. This is the product of an analytic torsion, indeed a half-density, coming from the Berezinian of the quadratic part times the perturbative expansion in Feynman diagrams.

21 We can make these choices up to a gauge transformation. For $\beta _0$, which is a constant map, there is no choice. The reason for the ħ factor, also present in [33], is that the term βB in the *J* action will contain a leading term $\hbar \,\xi \int _\Sigma B$. In the resulting observable, we will then have a leading factor $\exp {\textrm{i}}\xi \int _\Sigma B$. This is consistent with the usual definition of a Wilson loop as ${{\,\textrm{tr}\,}}_\rho P\exp {\textrm{i}}A$ with no ħ factor.

22 The integrals are over $C_k({\mathbb {R}}^2)$, the compactified configuration spaces of ${\mathbb {R}}^2$, where *k* is the number of vertices. In this case, also compactification at infinity is involved. Concretely, $C_k({\mathbb {R}}^2)$ may be defined as follows [18]. One first compactifies ${\mathbb {R}}^2$ to the sphere $S^2$, with ${\mathbb {R}}^2=S^2\setminus \{N\}$. Then one defines $C_k({\mathbb {R}}^2)$ as $\pi _{k+1}^{-1}(N)\subset C_{k+1}(S^2)$.

23 We have ignored signs and combinatorial factors. They all combine correctly because the formal BV computation precisely accounts for the principal-face contributions.

24 The only singular term would occur from the identification of fields joined by a propagator, which would leead to a “tadpole." This does not occur, however, since we assume the Lie algebra to be unimodular.

25 The compactified configuration spaces $C_k({\mathbb {R}}^4)$ are defined along the lines of footnote 22. The embedding σ then extends to a smooth map $C_k({\mathbb {R}}^2)\rightarrow C_k({\mathbb {R}}^4)$. For every *n*, we also have the map $C_{k+n}({\mathbb {R}}^4)\rightarrow C_k({\mathbb {R}}^4)$ that forgets the last *n* points. The compactified configuration space $C_{k,n}$ of n+k points in ${\mathbb {R}}^4$, *k* of which lie in the image of σ, is defined [18] as the fibered product of $C_k({\mathbb {R}}^2)$ and $C_{k+n}({\mathbb {R}}^4)$ over $C_k({\mathbb {R}}^4)$.

26 The form $\widetilde{\Theta }$ is a function of ħ and λ. At each order in ħ and λ, it is determined by the corresponding Feymnan diagrams. Most contributions actually vanish thanks to the Vanishing Lemmata in [66, Section 6.5.1].

27 This is in the same spirit as in the calculus of variations. More formally, we may consider $\mathcal {K}$ as the presheaf on the category of finite-dimensional smooth manifolds that assigns to *N* the set of smooth maps $N\times \Sigma \rightarrow M$ that are embeddings $\Sigma \rightarrow M$ for each point in *N*. A function *f* on $\mathcal {K}$ is then a natural transformation from $\mathcal {K}$ to the Yoneda embedding $\underline{{\mathbb {R}}}$ of ${\mathbb {R}}$. The differential ${\textrm{d}}f$ of *f* is just the composition of *f* with the canonical natural transformation $\underline{{\mathbb {R}}}$ to $\Omega ^1$, the latter being the functor that sends *N* to $\Omega ^1(N)$. Once *N* is chosen, the morphism $f_N$ assigns a smooth function on *N* to the map $N\times \Sigma \rightarrow M$, and ${\textrm{d}}f_N$ is just its differential on *N*. To show that *f* is locally constant, we just have to show that ${\textrm{d}}f_N=0$ for each *N*. The computation, for each *N*, reduces to the usual differentiation in finitely many variables.

28 We now also have new Feynman diagrams depending on λ. This introduces more potentially anomalous terms and consequently more terms in $\widetilde{\Theta }$, but the general structure is the same.

29 To work in lorentzian signature, we should use complex-valued fields.

30 To be more precise, we should first select background and residual fields. For simplicity, also of notation, we ignore this. Note that, working on $M={\mathbb {R}}^4$, we may assume background and residual fields to vanish.

31 In principle, we could use a different metric to define the Hodge star operator than the one used to split 2-forms and eventually appearing in the Yang–Mills action. However, this metric should anyway be chosen to be riemannian to get a Hodge decomposition (see also footnote 15).

32 The weight in the resulting functional integral is $\exp \left( \frac{{\textrm{i}}}{\hbar }\mathcal {S}_\text {YM}\right)$ even though we are in euclidean signature. We should see this as an analytic continuation of the euclidean functional integral for purely imaginary $\hbar \lambda$.

33 A different strategy would consist in finding first an observable $\widehat{W}_\gamma$ for *BF* theory whose BV pushforward is $W_\gamma$. The problem is that this observable is also highly nonlocal, so the product $\widehat{W}_\gamma \mathcal {O}_\sigma$ will likely also not be an observable.
